# Ribosomal protein eL39 is important for maturation of the nascent polypeptide exit tunnel and proper protein folding during translation

**DOI:** 10.1093/nar/gkac366

**Published:** 2022-05-27

**Authors:** Jelena Micic, Olga Rodríguez-Galán, Reyes Babiano, Fiona Fitzgerald, José Fernández-Fernández, Yunyang Zhang, Ning Gao, John L Woolford, Jesús de la Cruz

**Affiliations:** Department of Biological Sciences, Carnegie Mellon University, Pittsburgh, PA, USA; Instituto de Biomedicina de Sevilla, Hospital Universitario Virgen del Rocío/CSIC/Universidad de Sevilla, Seville, Spain; Departamento de Genética, Universidad de Sevilla, Seville, Spain; Instituto de Biomedicina de Sevilla, Hospital Universitario Virgen del Rocío/CSIC/Universidad de Sevilla, Seville, Spain; Departamento de Genética, Universidad de Sevilla, Seville, Spain; Department of Biological Sciences, Carnegie Mellon University, Pittsburgh, PA, USA; Instituto de Biomedicina de Sevilla, Hospital Universitario Virgen del Rocío/CSIC/Universidad de Sevilla, Seville, Spain; Departamento de Genética, Universidad de Sevilla, Seville, Spain; State Key Laboratory of Membrane Biology, Peking-Tsinghua Joint Centre for Life Sciences, School of Life Sciences, Peking University, Beijing, China; State Key Laboratory of Membrane Biology, Peking-Tsinghua Joint Centre for Life Sciences, School of Life Sciences, Peking University, Beijing, China; Department of Biological Sciences, Carnegie Mellon University, Pittsburgh, PA, USA; Instituto de Biomedicina de Sevilla, Hospital Universitario Virgen del Rocío/CSIC/Universidad de Sevilla, Seville, Spain; Departamento de Genética, Universidad de Sevilla, Seville, Spain

## Abstract

During translation, nascent polypeptide chains travel from the peptidyl transferase center through the nascent polypeptide exit tunnel (NPET) to emerge from 60S subunits. The NPET includes portions of five of the six 25S/5.8S rRNA domains and ribosomal proteins uL4, uL22, and eL39. Internal loops of uL4 and uL22 form the constriction sites of the NPET and are important for both assembly and function of ribosomes. Here, we investigated the roles of eL39 in tunnel construction, 60S biogenesis, and protein synthesis. We show that eL39 is important for proper protein folding during translation. Consistent with a delay in processing of 27S and 7S pre-rRNAs, eL39 functions in pre-60S assembly during middle nucleolar stages. Our biochemical assays suggest the presence of eL39 in particles at these stages, although it is not visualized in them by cryo-electron microscopy. This indicates that eL39 takes part in assembly even when it is not fully accommodated into the body of pre-60S particles. eL39 is also important for later steps of assembly, rotation of the 5S ribonucleoprotein complex, likely through long range rRNA interactions. Finally, our data strongly suggest the presence of alternative pathways of ribosome assembly, previously observed in the biogenesis of bacterial ribosomal subunits.

## INTRODUCTION

Ribosomes are highly complex ribonucleoprotein (RNP) machines comprised of two subunits that decode mRNA and catalyze protein synthesis in all organisms (1). In the yeast *Saccharomyces cerevisiae*, the small (40S) ribosomal subunit (r-subunit) contains a single, 18S ribosomal RNA (rRNA) and 33 ribosomal proteins (r-proteins), while the large (60S) r-subunit is composed of three rRNAs (5S, 5.8S and 25S) and 46 r-proteins (2,3).

In eukaryotes, assembly of rRNA and r-proteins into ribosomes occurs in three separate subcellular compartments, beginning in the nucleolus, continuing in the nucleoplasm, and concluding in the cytoplasm (2,4). Within the yeast nucleolus, the 35S pre-rRNA precursor to mature 18S, 25S and 5.8S rRNAs is transcribed by RNA polymerase I, and undergoes numerous covalent modifications, many of them directed by complementary small nucleolar RNAs (snoRNAs). External and internal spacer sequences are removed from pre-rRNAs by well-defined pathways that require a series of sequential endo- and exonucleolytic reactions (Supplementary Figure S1) (5–7). A precursor for 5S rRNA is transcribed separately by RNA polymerase III and is processed independently (5–7). Pre-rRNA modifications and processing occur concomitantly with the hierarchical assembly of r-proteins (8–12). In addition to pre-rRNAs and r-proteins, >300 different protein assembly factors (AFs) transiently associate with pre-ribosomal particles in a distinctive spatiotemporal manner, to ensure the speed, directionality and accuracy of r-subunit maturation (e.g. (4,10–15)).

Ribosomes contain different functional centers that enable protein synthesis, including the decoding center (DC) in the small r-subunit, and the peptidyl transferase center (PTC) and nascent polypeptide exit tunnel (NPET) in the large r-subunit (3). During translation, nascent polypeptide chains travel from the PTC through the NPET to exit from 60S r-subunits (16). This 90 Å long tunnel is comprised of rRNA from five out of six phylogenetically conserved secondary structure domains of the rRNAs of the large r-subunit, and three r-proteins: uL4 (yeast rpL4), uL22 (yeast rpL17) and eL39 (yeast rpL39) (Supplementary Figure S2) (3,17). Internal loops of uL4 and uL22 form the constriction sites of the NPET, and have been shown to be important for both assembly and function of ribosomes (18–23). The third protein that comprises the tunnel, eL39, an archaea- and eukaryote-specific r-protein, is located near the exit of the NEPT (3,24). Under laboratory conditions of growth, deletion of *RPL39* causes cold sensitivity (25–27), and results in decreased levels of 60S r-subunits (25,26,28). Ribosomes lacking eL39 display decreased translational accuracy and hypersensitivity to paromomycin, caused by an increased rate of binding of aminoacyl-tRNA to the A-site (25,29). In eubacteria, the long C-terminal extension of uL23 (bacterial rpL23, corresponding to yeast rpL25) is positioned adjacent to the NPET; this extension is replaced by eL39 in eukaryotes (30,31). Inclusion of eL39 in the NPET may explain differences between translation modes observed between prokaryotes and eukaryotes (31). The presence of eL39 in the NPET reduces the radius of the tunnel, which, together with the eukaryote-specific second constriction site (formed by the tip of the uL4 internal loop), may prevent external threats from entering the NPET and reaching the PTC (31). Interactions between a flexible tetraloop at the tip of helix H24 in 23S rRNA and eL39 in ribosomes from *Haloarcula marismortui* have been suggested to obstruct the tunnel exit and play regulatory roles during protein synthesis, ensuring a safe rate of translation (32).

We have previously studied the role of uL4 in construction of the NPET (19,33). Analysis of the yeast *rpl4Δ63–87* mutant, which lacks the uL4 internal loop that projects into the NPET and forms the second constriction site, reveals aberrant formation of the NPET that leads to deleterious effects on 60S r-subunit assembly (33). Improper maturation of the NPET prevents proper folding of several adjacent helices in domain IV (H68 and H69) and domain V (H74 and H75) of 25S rRNA (33). H69 is an integral component of the PTC, and also forms part of the intersubunit bridge B2a, which interacts with A and P-site tRNAs and connects the PTC and DC of the ribosome (3,34–36). Interestingly, eL39 is unable to stably associate with pre-ribosomal particles in the *rpl4Δ63–87* mutant, while assembly of uL22 is not affected (33). The failure to assemble eL39 in this mutant may be because particles are blocked in their maturation at a step before eL39 stably binds to them. Although it is still unclear how eL39 is loaded into pre-60S r-subunits, the absence of eL39 in pre-60S particles could explain some of the defects reported in the *rpl4Δ63–87* mutant.

We aim to understand in more detail the contribution of eL39 to ribosome biogenesis and function, especially how the absence of eL39 affects the maturation and function of the NPET. Our data suggest that eL39 assembles into pre-60S r-particles earlier than previously suggested. Most likely, eL39 is fully accommodated into the NPET during late nucleolar steps, but is initially loaded onto early pre-60S r-particles in an unstable binding state. We show that eL39 plays a role in the folding of nascent polypeptides, thus preventing protein aggregation. Our data also confirm that eL39 is required for 60S r-subunit biogenesis. Specifically, eL39 is required for efficient processing of both 27SB and 7S pre-rRNAs and for export of nascent particles from the nucleus to the cytoplasm. Our work reveals that 60S r-subunit assembly in the *rpl39Δ* mutant is delayed at the same step we previously reported for the *rpl4Δ63–87* mutant (33), as well as during earlier, middle nucleolar steps. Importantly, the absence of eL39 does not affect loading of the essential r-proteins uL4 and uL22 into pre-60S r-particles. Thus, changes observed in the *rpl39Δ* mutant are a direct consequence of the absence of eL39 from the NPET, and not an indirect effect caused by impairing the assembly of uL4 or uL22. Surprisingly, in the absence of eL39, we also observed the presence of normally middle-entering AFs in early pre-60S r-particles, and moderate but specific accumulation of normally early-associating and early-dissociating AFs in particles in middle stages of 60S r-subunit maturation. These data suggest that, in some instances, association and dissociation of AFs may occur ‘out of order’, indicating that these particles could represent parallel pathways during ribosome biogenesis, an observation previously reported during assembly of bacterial r-subunits (37–39).

## MATERIALS AND METHODS

### Strains and microbiological methods

All yeast strains used in this work are listed in Supplementary Table S1. Strains ORY211, ORY212, ORY251 and ORY312 were generated by PCR-based gene deletion-disruption of the *RPL39* gene in the strains W303 and ORY210, respectively. To this end, we amplified the *rpl39::natNT2* disruption cassette using the plasmid pAG25 (Supplementary Table S2) (40) as a template and the oligonucleotide pair L39Nat-up and L39Nat-low as primers (Supplementary Table S3). The PCR product was transformed into the strains W303, ORY210, Y1103 and Y965 by the lithium acetate method (41). Transformants were selected on YEPD medium containing 50 μg/ml nourseothricin, some candidates were chosen, and the correctness of the integration was verified by colony PCR.

To generate strains JWY10646 and JWY11985, the *rpl39::kanMX6* null allele was also constructed by homologous recombination of the PCR product containing kanMX6 plus sequences immediately upstream and downstream from the *RPL39* ORF. The plasmid used as a template was pFA6a-kanMX6 (Supplementary Table S2) (42). The PCR products were purified and transformed into the *BRX1-TAP* or *NOG2-TAP* strains from the Yeast TAP Collection (Horizon) (Supplementary Table S1). Candidates were selected on YEPD medium containing 200 μg/ml G418. Deletion was verified by colony PCR.

Strains expressing the eL39 C-terminally tagged with the Myc epitope (JWY11692 and JWY11694) were generated by replacing the stop codon of *RPL39* by the 13-Myc sequence in frame. The plasmid pFA6a-13Myc-kanMX6 (Supplementary Table S2) (42) was used to amplify the 13-Myc tag and sequences immediately upstream or downstream of the stop codon of the *RPL39* ORF. The PCR product was purified and transformed into the *SSF1-TAP* or *NOG2-TAP* strains (Horizon). Candidates were selected on YEPD medium containing 200 μg/ml G418. The presence of the C-terminal tag was confirmed by colony PCR, and the expression of the tagged protein was confirmed by western blotting using a monoclonal anti-Myc antibody (clone 9E10, Sigma-Aldrich).

To generate strains JDY1316 and JDY1317, a MATα derivative of ORY211 (*rpl39::*natNT2) was crossed to ORY77 (*trf4::*HIS3MX4) and ORY149 (*rrp6::*kanMX4), respectively. The resulting diploids were sporulated, and tetrads were dissected to obtain tetratypes.

Growth and handling of yeast strains and preparation of standard media were performed by established procedures (43). Rich medium (1% yeast extract, 2% peptone; YEP) or synthetic minimal medium (0.15% yeast nitrogen base, 0.5% ammonium sulfate; S) were supplemented with the appropriate amino acids and bases as nutritional requirements, and with 2% galactose (YEPGal and SGal, respectively), 2% glucose (YEPD and SD, respectively) or 2% raffinose (SRaf) as carbon sources. All solid media contained 2% agar. Unless otherwise indicated, yeast cells were grown at 30°C to an optical density at 600 nm (OD_600_) of about 0.8. AZC (azetidine-2-carboxylic acid), a drug that induces the misfolding of nascent proteins, was added to a final concentration of 2 mg/ml.

### Plasmids

The plasmids used in this study are listed in Supplementary Table S2. To generate YCplac33-RPL39 and YCplac111-RPL39, a PCR was performed using yeast genomic DNA as template and the oligonucleotides L39-up and L39-dw as primers (Supplementary Table S3), placed plus-minus 1 kb upstream and downstream from the start-stop codon of the *RPL39* ORF. The PCR product was cloned into pGEM^®^-T Easy Vector System (Promega); then, a ca. 2.5 kb EcoRI fragment was released and cloned into EcoRI-restricted YCplac33 and YCplac111 (44), respectively. To generate YCplac-111-RPL39-yEGFP, a PCR was performed using yeast genomic DNA as template and the oligonucleotides EcoRI-L39-up and L39-XbaI-dw as primers (Supplementary Table S3); then, the PCR product was digested with EcoRI and XbaI and cloned into EcoRI/XbaI-restricted YCplac-111-yEGFP. This construct generates a C-terminal fusion of the *RPL39* ORF with the coding region of yEGFP, which is expressed from the *RPL39* promoter. The remaining plasmids have been reported elsewhere (see Supplementary Table S2). More information about the construction of the different plasmids and the oligonucleotide primers that we used will be available upon request. *Escherichia coli* strain DH5α was used for cloning and propagation of plasmids. All recombinant DNA techniques were done according to established procedures (45).

### Sucrose gradient centrifugation

Cell extracts for polysome profile analyses were performed as described previously (46). Ten *A*_260_ units of total cell extract were loaded onto 7–50% sucrose gradients. These gradients were centrifuged at 39 000 rpm in a Beckman Coulter SW41 Ti rotor at 4°C for 2 h 45 min and fractionated using an ISCO UA-6 system with continuous monitoring at *A*_254_. To analyze the r-subunits specifically, 12 *A*_260_ units of total cell extract were loaded onto 7–50% sucrose gradients prepared in a low Mg^2+^-containing buffer (46). These gradients were also centrifuged at 39 000 rpm in a Beckman Coulter SW41 Ti rotor at 4°C but for 4 h 30 min.

When required, fractions of 0.5 ml were collected from the gradients. Protein and RNA were extracted from each fraction as previously described (47), and an equal volume of protein or RNA from individual fractions was subjected to western blot or northern blot analyses, respectively (see below).

### Western blot analysis and antibodies

Total yeast protein extracts were prepared and analyzed by western blotting according to standard procedures (45). Commercial (anti-GFP, Roche; anti-Myc, Sigma-Aldrich; anti-Nop2, Thermo Fisher Scientific) and specific primary antibodies against r-proteins and AFs were used. Secondary goat anti-mouse or anti-rabbit horseradish peroxidase-conjugated antibodies (Bio-Rad), or alkaline-phosphatase-conjugated anti-mouse or anti-rabbit secondary antibodies (Promega) were used. Proteins were detected using a chemiluminescence detection kit (Super-signal West Pico, Pierce) and a ChemiDoc MP™ system (Bio-Rad). Alternatively, colorimetric detection was performed using NBT and BCIP (Promega).

### Pulse-chase assays of pre-rRNA

Pulse-chase labelling of pre-rRNA was performed exactly as previously described (48), using 100 μCi of [5,6-^3^H]uracil (45 to 50 Ci/mmol; Perkin Elmer) per 40 OD_600_ units of yeast cells. Cells were first transformed with an empty YCplac33 plasmid (*CEN URA3*) (Supplementary Table S2) to make them prototrophic for uracil. Transformants were grown in liquid SD-Ura medium at 22°C to exponential growth phase, pulse-labelled for 2 min with [5,6-^3^H]uracil, and chased for 5, 15, 30 and 60 min with SD medium containing an excess of non-radioactive uracil (1 mg/ml) also at 22°C. Total RNA was extracted by the acid-phenol method (49). Radioactive uracil incorporation was measured by scintillation counting. Normally, 20 000 cpm per RNA sample were loaded and resolved on 1.2% agarose-6% formaldehyde and 7% polyacrylamide–8 M urea gels (50). Then, RNA was transferred to Hybond-N nylon membranes (Amersham). Membranes were dried, UV cross-linked, sprayed with EN^3^HANCE (Du Pont) and dried again before exposing them to X-ray films for 4 days at –80°C with an intensifying screen (48).

### Northern hybridization and primer extension analyses

Steady-state levels of pre- and mature rRNAs were assessed by northern and primer extension analyses as described previously (48,50). Specific oligonucleotides, whose sequences are provided in Supplementary Table S3, were 5′-end labelled with 20 μCi of [γ-^32^P]ATP (6000 Ci/mmol; Perkin Elmer) and used as probes or primers. In all experiments, total RNA was extracted from samples corresponding to 10 OD_600_ units of exponentially grown cells. Equal amounts of total RNA (5 μg) were loaded and resolved on 1.2% agarose–6% formaldehyde and 7% polyacrylamide–8 M urea gels. Then, RNA was transferred to and immobilized on Hybond-N nylon membranes and subjected to hybridization (48,50). Primer extension was done with the same RNA samples as those used for northern blot analysis according to Venema *et al.* (50). The resulting cDNA products were resolved on 6% polyacrylamide–8 M urea gels, which were then dried on Whatman 3MM filter paper. Both membranes and filter papers were subjected to phosphorimager analysis with a Typhoon™ FLA9000 imaging system (GE Healthcare).

### Fluorescence microscopy

To visualize nucleolar release and nuclear export of assembling 60S r-subunits, strains lacking eL39 were transformed with the pRS316-RPL25eGFP/mRFPNOP1 reporter plasmid (a gift from J. Bassler and E. Hurt), grown in SD-Ura at 30°C, and shifted to 16°C for 5 h. Cells were anchored to MaTek plates using 20–30 μl of 1 mg/ml concanavalin A (ConA, Fisher Scientific). The ConA was dried on plates for 30–45 min before adding cells. Cell images were obtained with a Zeiss LSM 880 laser scanning confocal microscope at 600× magnification. Images were acquired using ZEN software (blue edition, by Zeiss) and the images were processed using the Fiji software for Mac OS X (National Institutes of Health).

To address the timing of assembly of eL39, an *rpl39*Δ strain was co-transformed with plasmids YCplac111-RPL39-eGFP, pRS314-NOP1mRFP, and either pRS316-*GAL*-*NMD3*Δ*100* or pRS316-*GAL*-*NMD3FL* (gifts from A. Jacobson), which express a dominant-negative truncation or a wild-type allele of the *NMD3* gene, respectively, under the control of an inducible *GAL* promoter (51). Transformants were grown in selective SRaf medium at 30°C and shifted to selective SGal medium at 30°C to induce the expression of the Nmd3 proteins. Culture aliquots were washed, and resuspended in sterile distilled water before cells were examined with an Olympus BX61 fluorescence microscope equipped with a digital camera; images were analyzed using the CellSens software (Olympus) and processed using Adobe Photoshop CC (Adobe Systems, Inc.).

### Affinity purification of L39-GFP containing pre-ribosomal particles

Complexes containing GFP-tagged eL39 protein were precipitated following the one-step GFP-Trap A procedure (Chromotek) with GFP-Trap A beads, exactly as described in (52). The proteins from the purified complexes were extracted by boiling the beads with Laemmli buffer and analyzed by western blotting (53). Pre-rRNAs and mature rRNAs were recovered from the beads by phenol-chloroform extraction and assayed by northern hybridization as indicated above.

### Affinity purifications of assembling 60S r-subunits using TAP-tagged AFs

Pre-60S r-particles were affinity-purified from whole-cell extracts with magnetic Dynabeads (Thermo Fisher Scientific), using TAP-tagged AFs Ssf1, Brx1 or Nog2, as previously described (54,55) with the following modifications. Cultures (250–500 ml) were grown in liquid YEPD medium to an OD_600_ of 0.7–0.9. Cells were resuspended in 4 ml of lysis buffer (50 mM Tris–HCl, pH 7.5, 150 mM NaCl, 10 mM MgCl_2_, 0.075% Triton X-100), and subjected to vortexing with glass beads (0.5 mm diameter, Biospec Products) eight times for 30 s, with incubation on ice in between vortexing. Extracts were clarified by centrifugation and bound to IgG-coated Dynabeads at 4°C for 1 h. Beads were washed three times with the lysis buffer, and pre-60S r-subunits were eluted by cleaving the TEV protease site within the TAP-tag, using 1–2 μl of TEV Protease (Thermo Fisher Scientific). AFs and r-proteins from assembling r-subunits were precipitated with 10% trichloroacetic acid (TCA), resuspended in SDS sample buffer and separated by SDS-PAGE on 4–20% Tris–glycine Novex gels or NuPage 12% Bis–Tris gels (Thermo Fisher Scientific).

### Analysis of pre-60S r-subunits by semi-quantitative mass spectrometry (iTRAQ)

Pre-ribosomal particles were purified as above, with the following modifications. Cell pellets were resuspended in TNM150 buffer (50 mM Tris–HCl, pH 7.5, 150 mM NaCl, 1.5 mM MgCl_2_, 0.1% NP-40 and 5 mM 2-mercaptoethanol (Sigma-Aldrich)), and cell lysates were prepared using glass beads. Lysates were incubated with IgG-coated Dynabeads at 4°C for 1 h; NP-40 was omitted from the buffer in all succeeding steps. Purified samples were digested with trypsin and 8-plex labeled with iTRAQ reagents 113, 114, 115, 116, 117, 118, 119 and 121 (Applied Biosystems) in the Penn State Hershey Core Research Facilities. Peptides were separated by two-dimensional (2D) liquid chromatography, and parent ions were identified on a Sciex 5600 liquid chromatography mass-spectrometer system. Protein Pilot 5.0 was used to obtain iTRAQ ratios as an average of all peptides for each protein. Proteins identified with >99.9% confidence were used for further data analysis. Data were normalized to the TAP-tagged AFs Brx1 and Nog2.

### Analysis of aggregated proteins

Isolation of aggregated proteins was done from whole cell extracts of yeast W303, ORY211 and JDY532 strains exactly as described in (56). Briefly, 40 OD_600_ units of cells, grown to mid-log phase in YPD, were harvested, washed with water containing 15 mM NaN_3_, and resuspended in 0.5 ml of freshly prepared lysis buffer (20 mM sodium phosphate, pH 6.8, 10 mM DTT, 1 mM EDTA, 0.1% Tween 20) supplemented with 1x Complete EDTA-free protease inhibitor cocktail (Roche), 3 mg/ml zymolyase T20 (USBiological) and 1.25 U/ml benzonase (Merck). Samples were incubated at room temperature for 15 min, chilled on ice for 5 min, and tip sonicated (Branson sonifier 450; three times 10 s at duty cycle 40%). Upon sonication, the lysates were cleared by centrifugation at 200 × *g* for 20 min at 4°C, and the supernatants were adjusted to an identical protein concentration of 10 mg/ml. Supernatant aliquots of 20 μl were taken and boiled with 40 μl of SDS/Laemmli sample buffer (total extract samples). Then, the supernatants were centrifuged at 16 000 × *g* for 20 min at 4°C to pellet the aggregated proteins. After removing the supernatants, the pellets were washed twice with 0.5 ml of wash buffer (20 mM sodium phosphate, pH 6.8, 1× Complete EDTA-free protease inhibitor cocktail) containing 2% NP-40 (WB-NP); then, pellets were resuspended in 0.5 ml WB-NP, sonicated (10 s at duty cycle 40%), and centrifuged at 16 000 × *g* for 20 min at 4°C. Pellets were again washed twice with 0.5 ml of wash buffer and then resuspended in wash buffer, sonicated, and centrifuged as above. Pellets were resuspended in 50 μl of SDS/Laemmli sample buffer and boiled (protein aggregate samples). Total extract and protein aggregate samples were then separated by 4–12% gradient SDS-PAGE and analyzed by colloidal blue Coomassie staining and western blotting with selected antibodies.

### Inspection of ribosome structures

Images of 60S r-subunit assembly intermediates were visualized using the UCSF Chimera 1.14.0 program (57) from PDB files 6EM1, 6EM3, 6EM4,6EM5, 6ELZ (10), 6YLX, 6YLY (58), 3JCT (59), 5FL8 (60) and 5H4P (61).

## RESULTS

### Deletion of *RPL39* causes a severe growth defect and affects production of 60S r-subunits

To expand our understanding of the involvement of eL39 in translation, and to investigate its importance during assembly of 60S r-subunits, we used an *rpl39Δ* yeast mutant in the W303 background in which the entire *RPL39* ORF was replaced by a natNT2 marker, or in the BY4743 background in which the entire *RPL39* ORF was replaced by a kanMX4 marker. Successful deletion was confirmed by western blotting using whole cell extracts from *RPL39* and *rpl39Δ* cells (Supplementary Figure S3A). Consistent with previously published data (25,26,62), the *rpl39Δ* mutant exhibited an almost lethal phenotype at 16°C and very slow growth at 22°C (Supplementary Figure S3B). These findings are independent of the yeast genetic background, since similar results were observed for the W303- and BY4743-derived *rpl39Δ* mutants (Supplementary Figure S3C).

To investigate how the absence of eL39 affects the synthesis and maturation of 60S r-subunits, we performed polysome profile analysis with lysates from the *rpl39Δ* and *RPL39* strains grown at 22, 30 or 37°C. The absence of eL39 caused a deficit in mature 60S r-subunits, evident by decreased levels of total 60S versus total 40S r-subunits. In addition, halfmers (polysomes containing one 40S r-subunit bound to the start codon in mRNAs without a partner 60S r-subunit) were also detected at all temperatures, further confirming a shortage of 60S r-subunits (Supplementary Figure S4A). Quantification of total r-subunits using low-Mg^2+^ run-off sucrose gradients showed a 20–30% reduction of total 60S relative to 40S r-subunits in the *rpl39Δ* mutant compared to its isogenic wild-type counterpart (Supplementary Figure S4B). These results are in agreement with previously published data (25–28). The observed growth and polysome profile defects were fully reversed by expression of wild-type *RPL39* from a plasmid at each temperature (Supplementary Figure S5).

### The absence of eL39 leads to inefficient folding of nascent polypeptides and increased protein aggregation

Since eL39 is a component of the NPET, we first sought to investigate the effect of the absence of eL39 during translation. Nascent polypeptides undergo folding co-translationally as they emerge from the NPET. This process is assisted by different chaperone systems, such as the nascent chain-associated complex (NAC), the Hsp70 chaperone SSB, the ribosome-associated complex (RAC), the prefoldin complex, and the chaperonin TriC/CCT complex (reviewed in (63–65)). Some of these complexes interact with nascent polypeptide chains at the exit of the NPET in 60S r-subunits, where eL39 is located (reviewed in (66)). Thus, we wanted to investigate whether the absence of eL39 alters the function of these chaperones with ribosomes and affects protein folding.

**Figure 1.**
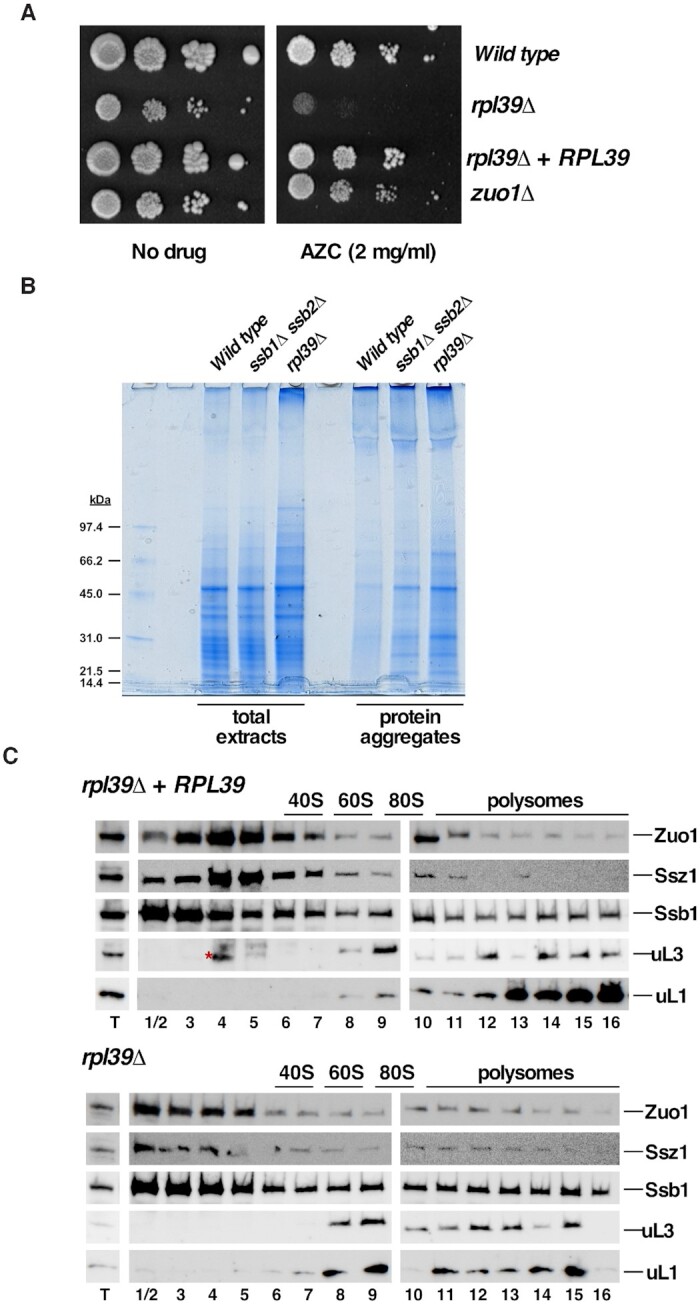
Deletion of *RPL39* increases protein misfolding and aggregation. (**A**) Deletion of *RPL39* increases sensitivity to misfolding of newly translated proteins induced by incorporation of azetidine-2-carboxylic acid (AZC). The ORY211 strain was transformed with either the empty plasmid YCplac111 (*rpl39Δ*) or the YCplac111-*RPL39* plasmid (*rpl39Δ* + *RPL39*). Cells were grown in liquid SD-Leu medium, diluted to an OD_600_ of 0.05 and spotted in 10-fold serial dilutions onto SD-Leu plates without (no drug) or supplemented with 2 mg/ml AZC (AZC). Plates were incubated for 4 days at 30°C. As controls, isogenic W303-1A (*Wild type*) and JDY509 (*zuo1Δ)* strains, also transformed with YCplac111, were used. (**B**) Analysis of protein aggregation in the *rpl39Δ* cells. Strains W303-1A (*Wild type*), JDY532 (*ssb1Δ ssb2Δ*) and ORY211 (*rpl39Δ)* were grown to log phase in YEPD medium at 30°C. Total protein extracts and protein aggregates were prepared, separated by SDS-PAGE and visualized by Coomassie staining. (**C**) eL39 is dispensable for stable interaction of SSB-RAC with ribosomes. The above *rpl39Δ*, and *rpl39Δ* + *RPL39* strains were grown in SD-Leu at 30°C to an OD_600_ of around 0.8. Cells were harvested and total cell extracts were prepared. Ten *A*_260_ units of each extract were resolved on 7–50% sucrose gradients. Sedimentation is shown from left to right. The sedimentation positions of free 40S and 60S r-subunits, 80S vacant ribosomes/monosomes and polysomes are indicated. Fractions were collected from the gradients and the proteins were extracted from equal volumes of each fraction, resolved on SDS-PAGE gels and subjected to western blotting using specific antibodies against the indicated proteins. T stands for total extract and numbers correspond to fraction numbers. The red asterisk in the upper fractionation indicates a background cross-reacting band in fractions 4 and 5.

First, we observed that deletion of *RPL39* causes hypersensitivity to the misfolding-inducing drug azetidine-2-carboxylic acid (AZC) (Figure 1A). AZC is a proline analogue that incorporates into proteins competitively with proline and interferes with *de novo* protein folding (67). Interestingly, the sensitivity of the *rpl39Δ* mutant is evident even at a concentration that is insufficient to completely inhibit the growth of a strain lacking Zuo1, a subunit of the RAC complex that has been previously reported to be sensitive to this drug ((67); see also Figure 1A). Second, since ribosome-associated chaperones prevent protein aggregation by assisting proper folding (68,69), we examined whether the absence of eL39 enhanced protein aggregation. To do so, total protein extracts and protein aggregates were prepared from *rpl39Δ* and wild-type cells, separated by SDS-PAGE and visualized by Coomassie staining. An *ssb1Δ ssb2Δ* strain was used as a positive control (68). As shown in Figure 1B, general protein aggregation was comparable or even slightly higher in the *rpl39Δ* mutant than in the positive control, suggesting the involvement of eL39 in preventing protein aggregation.

Finally, to directly investigate whether the absence of eL39 negatively impacts binding of chaperone complexes to ribosomes, we performed sucrose gradient fractionation of lysates from the *rpl39Δ* strain and its wild-type counterpart, followed by western blot analysis to reveal the sedimentation patterns for SSB and RAC. In yeast, RAC has been shown to contact eL31 (yeast rpL31) (66) and SSB contacts eL39 (70), while the combined absence of eL31 and eL39 in yeast is lethal (26). In the presence of eL39, these complexes co-migrated with r-subunits, but a significant portion also sedimented at the top of the gradients, as free complexes. However, in the absence of eL39, the sedimentation patterns of components of these two chaperone complexes did not change significantly (Figure 1C), suggesting that eL39 is not critical for stable interaction of SSB-RAC with translating ribosomes. Whether or not eL39 is necessary to induce the activity of these ribosome-associated complexes still remains unexplored.

In summary, we conclude that eL39 prevents protein misfolding and therefore protein aggregation, similarly to what has been previously observed for the ribosome-associated SSB-RAC chaperone system (66,71,72). Many mutants that affect ribosome biogenesis exhibit these phenotypes (56,73). Thus, whether the increased protein misfolding and aggregation phenotypes observed in the *rpl39Δ* strain are specific features of this mutant or the mere consequence of its ribosome biogenesis defect remains to be understood.

### The absence of eL39 perturbs processing of 27SB and 7S pre-rRNAs and affects nuclear exit of maturing 60S r-subunits

**Figure 2.**
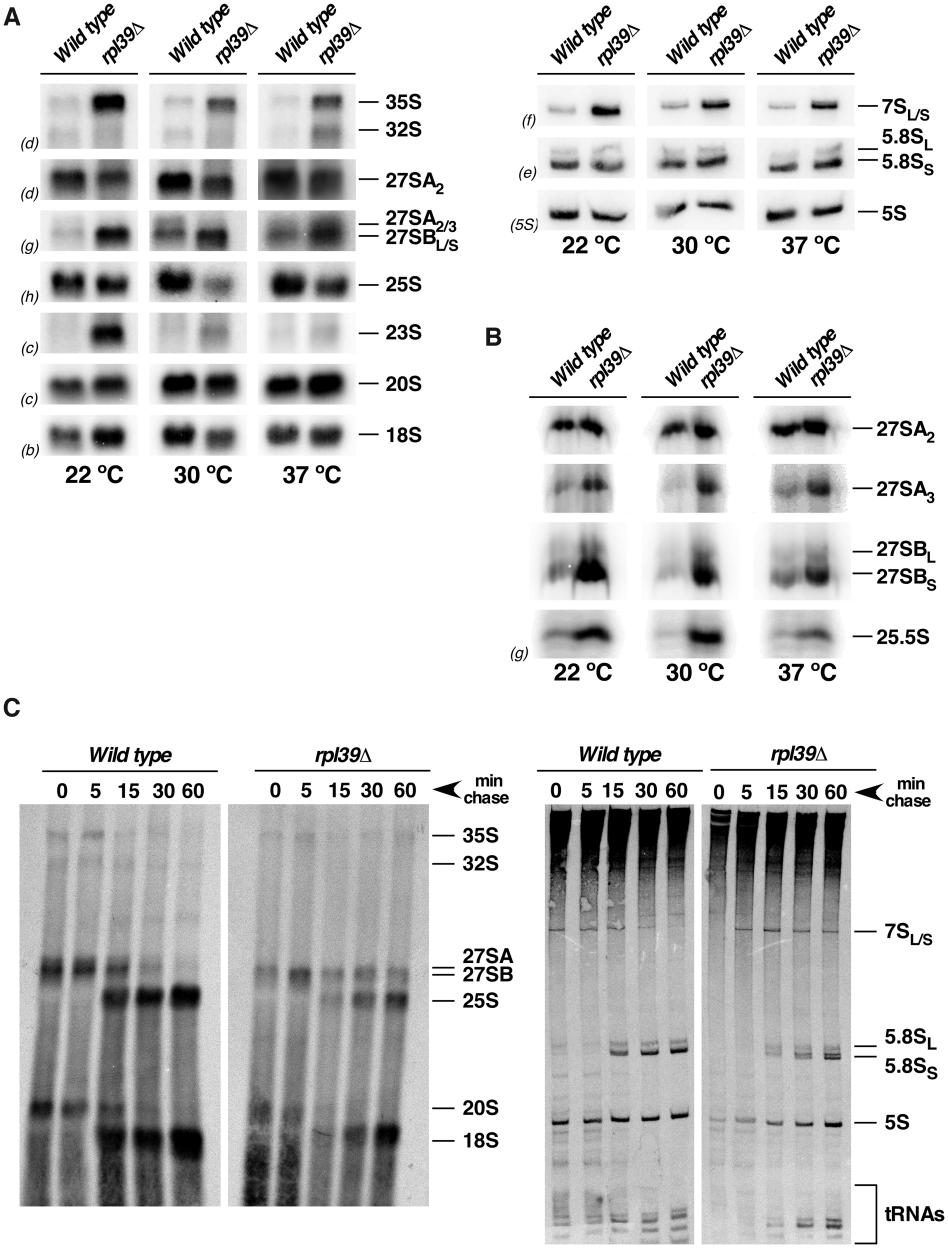
The absence of eL39 delays processing of 27SB and 7S pre-rRNAs. The isogenic W303-1A (*Wild type*) and ORY211 (*rpl39Δ*) strains were grown in YEPD to an OD_600_ of around 0.8 at 22, 30 and 37°C. Cells were harvested and total RNA was extracted. Equal amounts of RNA (5 μg) were subjected to northern hybridization or primer extension analysis. (**A**) Northern analysis of high-molecular-mass pre-rRNAs and mature rRNAs (left). Northern analysis of low-molecular-mass pre-rRNAs and mature rRNAs (right). (**B**) Primer extension analysis with probe g within ITS2. This probe allows detection of 27SA_2_, 27SA_3_, both 27SB_L_ and 27SB_S_ pre-rRNAs and 25.5S species. Probes (in parentheses) are described in Supplementary Figure S1A and Table S3. (**C**). Pulse-chase analysis. The *rpl39Δ* strain was transformed with the YCplac33-*RPL39* (wild-type) or the YCplac33 (*rpl39Δ)* plasmids and grown in SD-Ura to an OD_600_ of around 0.8 at 30°C. Cells were pulse-labelled for 2 min with [5,6-^3^H]uracil and then chased for 5, 15, 30, and 60 min with an excess of unlabeled uracil. Total RNA was extracted, and samples (20 000 cpm per sample) were loaded and separated on a 1.2% agarose-6% formaldehyde gel (left) or a 7% polyacrylamide–8 M urea gel (right), transferred to nylon membranes, and visualized by fluorography. The positions of the different pre-rRNAs and mature rRNAs are indicated. Note that the doublet observed for the mature 5.8S rRNA species in the samples from the *rpl39Δ* strain is the consequence of a technical problem and not a biological result.

To examine whether and how the absence of eL39 affects pre-rRNA processing during maturation of pre-60S r-subunits (see Supplementary Figure S1), we assayed changes in steady-state levels of pre- and mature rRNAs in isogenic *rpl39Δ* mutant and wild-type strains grown at 22, 30 and 37°C, by northern hybridization and primer extension. Significant accumulation of both 27SB_L_ and 27SB_S_ pre-rRNAs was observed upon deletion of *RPL39* at all temperatures, and amounts of mature 25S rRNA decreased (Figure 2A, left, and B). Analysis of low-molecular-mass rRNAs revealed a marked increase in steady-state levels of 7S pre-rRNAs but no alteration in the levels of mature 5.8S and 5S rRNAs (Figure 2A, right). The increase of the primer extension stop at site C_2_ indicated that 25.5S pre-rRNA also accumulated at all tested temperatures. In addition, 35S and 23S pre-rRNAs also accumulated; this was likely a secondary effect of the inefficient recycling of early AFs (e.g. (74)) or the conversion from co- to post-transcriptional pre-rRNA processing in stressed *rpl39Δ* cells (e.g. (75–77)).

We also assayed the synthesis and turnover of pre-rRNA by [5,6–^3^H]uracil pulse-chase labelling experiments. In the *rpl39Δ* cells at 30°C, we observed delayed processing of 35S and 27S pre-rRNAs. The 27SB pre-rRNAs could still be detected 60 min after chase, and much less labeled mature 25S rRNA was detected. Processing of 20S pre-rRNAs was only slightly impaired (Figure 2C, left). Analysis of low-molecular-mass RNAs showed a delay in processing of the 7S pre-rRNAs to 5.8S rRNAs, while synthesis of 5S rRNA was not significantly affected (Figure 2C, right).

To investigate effects of the absence of eL39 on intracellular trafficking of pre-60S r-subunits, we analyzed localization of the 60S r-subunit reporter uL23-yEGFP in both the *rpl39Δ* and *RPL39* strains. Consistent with a block in processing of 27SB and 7S pre-rRNA intermediates, we observed accumulation of uL23-yEGFP in the nucleolus and the nucleoplasm of *rpl39Δ* cells grown at 30°C, which was enhanced at low temperatures (13°C) (Supplementary Figure S6). Thus, the absence of eL39 affected the exit of maturing pre-60S r-particles from the nucleolus to the nucleoplasm, and subsequent export of them to the cytoplasm, especially at low temperatures.

Together, these results strongly suggest that, in the absence of eL39, pre-60S r-particles that contain 27SB and 7S pre-rRNAs are accumulating during late nucleolar and early nucleoplasmic steps of 60S r-subunit maturation.

### eL39 associates with pre-60S r-particles during middle nucleolar stages of 60S r-subunit biogenesis

Previous analysis of cryo-electron microscopy (cryo-EM) structures indicated that eL39 first stably associates with pre-60S r-particles transitioning from the nucleolus to the nucleoplasm (Nog2 particles) ((58,59); see Supplementary Figure S7). This is in agreement with the almost complete absence of density for eL39 in preceding nucleolar pre-60S intermediates, which correspond to cryo-EM particles from states E, NE1 and NE2 ((10,58); Supplementary Figures S7 and S8). However, the lack of density of any given protein as visualized by cryo-EM does not preclude the possibility of its association with particular particles. For example, molecules may bind less stably and thus can be lost during affinity purification of pre-60S r-particles. Also, they may stably bind to pre-rRNA to form a complex that has not yet fully accommodated into its stable conformation in pre-60S r-subunits, and thus remain flexible and not detectable by cryo-EM. As the absence of eL39 resulted in a block in maturation of particles containing 27SB pre-RNAs, which includes at least eight different cryo-EM intermediates (particles from states A-E, NE1 and NE2, and Nog2 state 1) ((10,58); Supplementary Figure S7), we sought to investigate in more detail the timing of incorporation of eL39 into pre-60S r-particles.

We took three approaches to map the assembly of eL39 into ribosomes. First, we tested the localization of almost fully functional eL39-yEGFP in a *NMD3Δ100* dominant-negative mutant that blocks nuclear export of pre-60S r-particles (78). Under conditions of basal expression of the Nmd3Δ100 protein, both uL23-yEGFP, which was used as a control for nucleolar assembly (79,80), and eL39-yEGFP localized to the cytoplasm. However, upon overexpression of the Nmd3Δ100 protein, both GFP-tagged r-proteins accumulated in both the nucleolus and the nucleoplasm, partially colocalized with the nucleolar marker Nop1-mRFP (Supplementary Figure S9). These results indicate that eL39 stably assembles into nucleolar particles.

**Figure 3.**
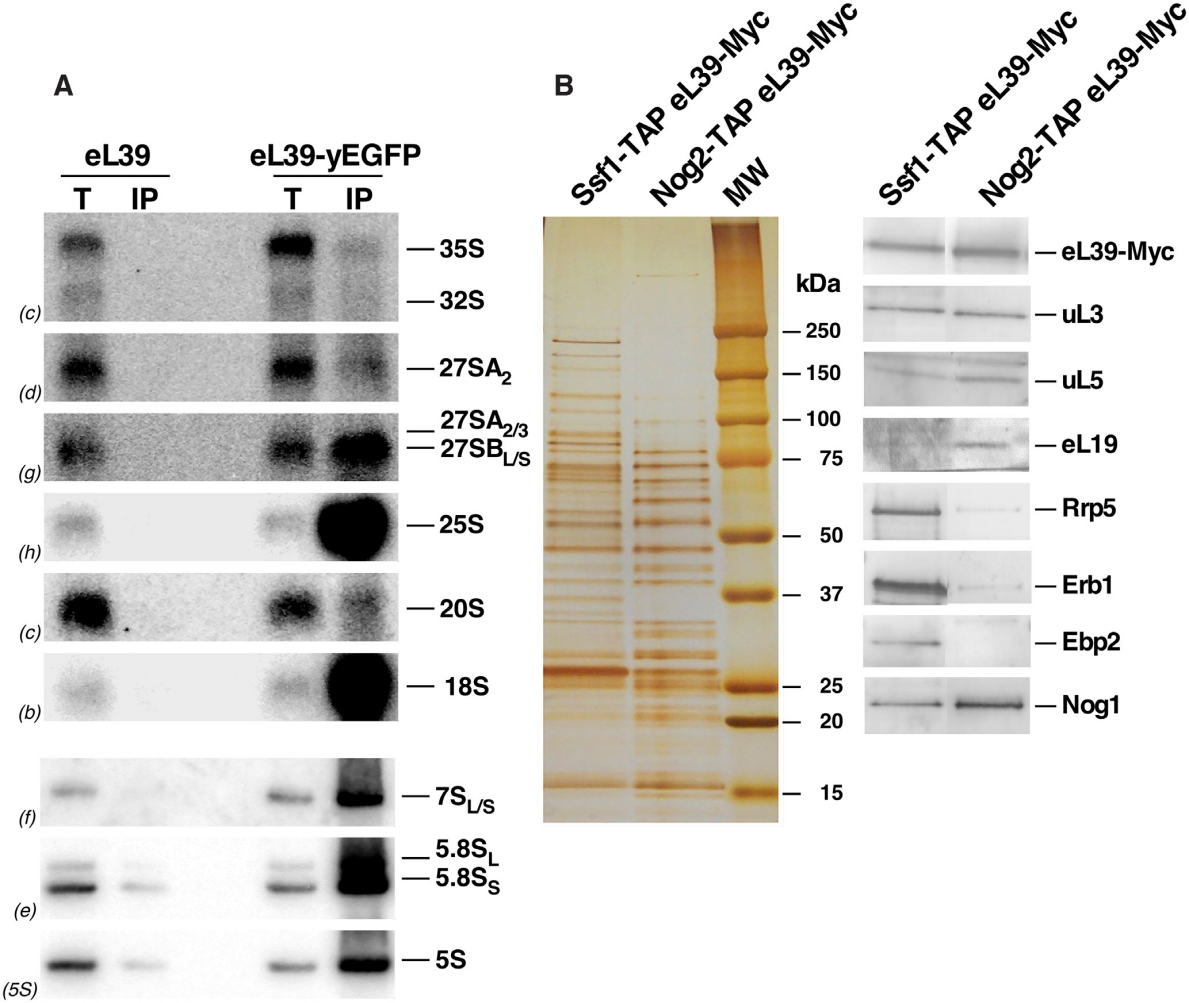
eL39 is stably accommodated into early and medium pre-60S ribosomal particles. (**A**) eL39 was affinity-purified with GFP-Trap^®^_A beads, using total cell extracts of ORY211 (*rpl39Δ*) cells expressing eL39-yEGFP or untagged eL39 (as a negative control). Total RNA was extracted from whole cell extracts (T) and immunoprecipitates (IP), and analyzed by northern blotting. The indicated probes (in parentheses) were used to detect the different rRNA species (Figure S1A, Table S3). (**B**) Pre-60S r-subunit assembly intermediates were purified via TAP-tagged AFs Ssf1 and Nog2. The protein composition of the purified particles was analyzed by SDS-PAGE followed by silver staining (left) and western blotting with antibodies against the indicated r-proteins and AFs (right).

Second, we affinity purified eL39-yEGFP containing complexes and determined which pre-rRNA intermediates co-purified with eL39-yEGFP by northern hybridization. As expected for a r-protein, GFP-tagged eL39 co-purified with mature 25S, 5.8S and 5S rRNAs (Figure 3A). Mature 18S rRNA also co-purified, which likely reflects translating ribosomes, or the common non-specific binding of 40S and 60S r-subunits in Mg^2+^-containing buffers (52). Importantly, eL39-yEGFP co-purified with significant amounts of 27SB and 7S pre-rRNAs, but not 35S, 27SA_2_ and 20S pre-rRNAs (Figure 3A). These results were specific, as only background levels of pre- and mature rRNAs were co-purified with untagged eL39 protein. These data indicate that eL39 stably assembles into nucleolar particles containing 27SB pre-rRNAs (for particles see Supplementary Figure S7).

Third, to investigate with which 27SB-containing particles eL39 first associates, we Myc-tagged eL39 in *SSF1-TAP* and *NOG2-TAP* strains, affinity purified associated r-particles and performed western blotting with a set of antibodies. Ssf1 is an early entering assembly factor thought to associate with pre-RNA co-transcriptionally, and to exit assembling particles between states C and D (10,81–83). Nog2 particles were used as a control, since eL39 has been found stably bound to these particles (59). As expected, we observed significantly higher levels for selected nucleolar factors (Rrp5, Erb1 and Ebp2) in Ssf1-containing particles than in Nog2-containing particles, since these AFs assemble early, and are thought to exit pre-ribosomes before Nog2 enters (Figure 3B, see Discussion). Amounts of uL3, an early assembling r-protein (9,80,84,85), were comparable between the two affinity-purified samples, as expected for an r-protein that does not dissociate from r-subunits once stably assembled. On the other hand, levels of late-stabilizing eL19 protein (10,86) were higher in Nog2-containing particles (Figure 3B). Strikingly, we detected significant amounts of eL39-Myc protein in Ssf1-containing particles, comparable to the amount in Nog2-containing particles. Thus, these results indicate that eL39 likely associates with maturing pre-60S r-particles early during biogenesis, at a stage that includes state C or even earlier intermediates (Supplementary Figure S7) but cannot be visualized by cryo-EM until after it has stably associated with the NPET in the nucleolar Nog2 state 1 pre-60S particles.

### The absence of eL39 blocks pre-60S maturation upstream from rotation of 5S RNP but does not affect assembly of r-proteins uL4 and uL22

Since portions of both uL4 and eL39 are located in the NPET, we wanted to test whether the absence of eL39 causes a phenotype similar to that observed in the *rpl4Δ63–87* mutant. Previous characterization of the *rpl4Δ63–87* mutant, using AF Nog2 as a bait, uncovered a block in pre-60S r-particle maturation that affects rotation of the 5S RNP (33,87). Levels of the ATPase Rea1, the Rix1 complex (Ipi1, Rix1/Ipi2 and Ipi3), and Sda1 decreased significantly in the particles containing uL4Δ63–87 truncated r-protein. In addition, AFs involved in nucleo-cytoplasmic export of assembling 60S r-subunits, Bud20, Arx1 and Alb1, also decreased. To study the effects of the absence of eL39 on assembly of the 60S r-subunits, we used two AFs as baits: Brx1, an early associating AF that exits the assembly pathway during the transition from state E to NE1 particles (10,58,88,89) and Nog2, present in late-nucleolar and early nucleoplasmic assembling 60S r-subunits (59,90) (see Supplementary Figure S7).

**Figure 4.**
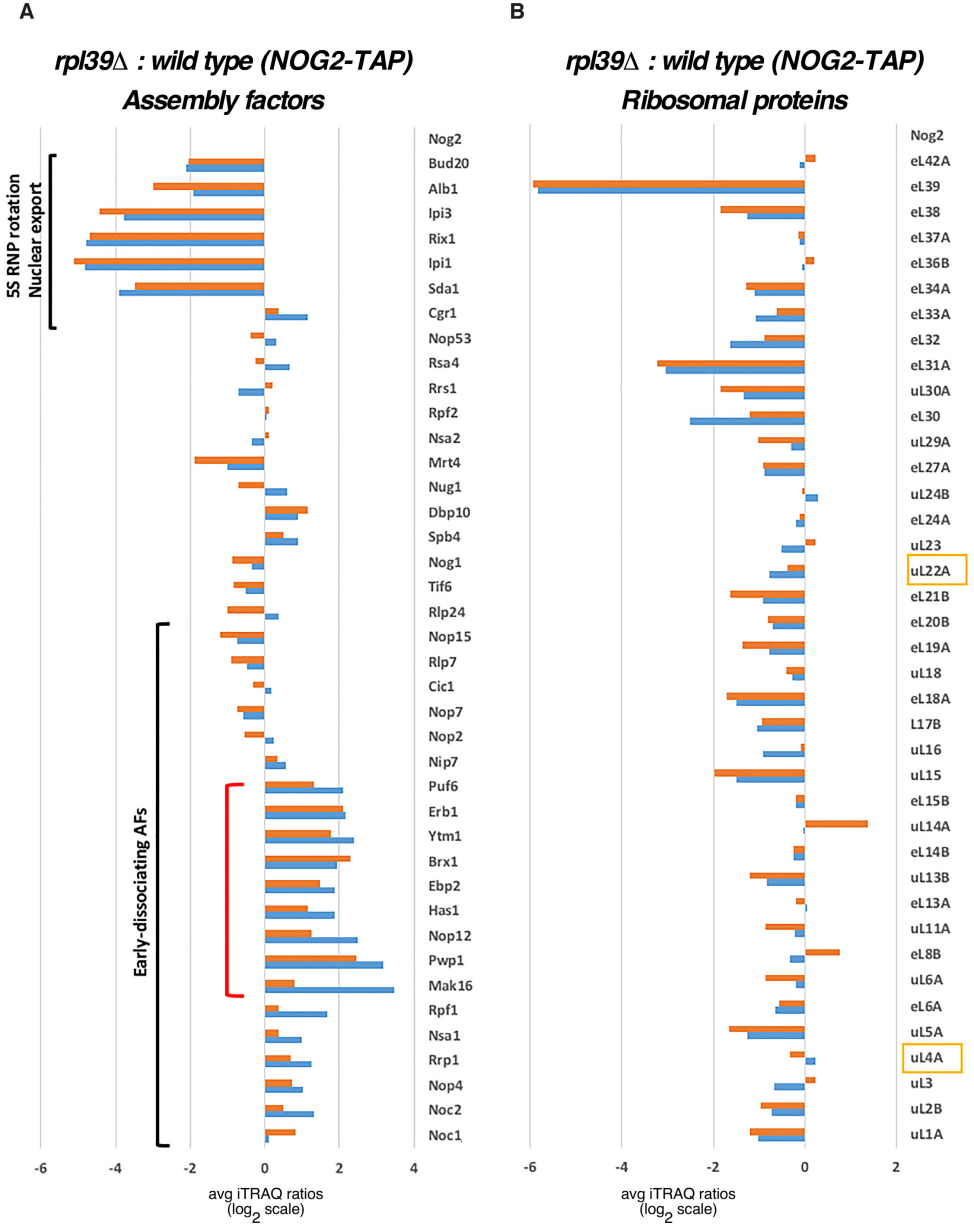
eL39 is important for maturation of pre-60S r-particles in both early and middle stages of assembly. Wild-type (JWY11983) and *rpl39Δ* (JWY11985) strains expressing a TAP-tagged version of Nog2 were grown in YEPD at 30°C and shifted to 16°C for 5 h. Assembling 60S r-subunits were affinity-purified and samples were used for iTRAQ to quantify relative changes in levels of (**A**) AFs, and (**B**) r-proteins in the *rpl39Δ* mutant compared to wild-type cells. The ratios were normalized to levels of the bait protein Nog2. The fold-change is shown using bar graphs in log_2_ scale. Blue and red bars represent two biological replicates. AFs that disassociate from the assembly pathway during early stages of assembly, and AFs involved in 5S RNP rotation and nuclear export of pre-60S r-particles are labelled with black brackets. The red bracket represents a subset of early AFs that accumulates in the *rpl39Δ* mutant strain. The other two components of the NPET besides eL39, r-proteins uL4 and uL22, are framed in yellow.

First, we affinity-purified pre-60S r-particles, using Nog2 as a bait, from *RPL39* and *rpl39Δ* cells shifted from 30°C to 16°C for 5 h. Then, we examined changes in the protein constituents of purified particles by semi-quantitative mass spectrometry (iTRAQ). Comparable to the results obtained for the *rpl4Δ63–87* mutant, we observed a significant decrease in levels of AFs involved in rotation of the 5S RNP in Nog2-containing particles from *rpl39Δ* cells as well as Bud20 and Alb1 (Figure 4A). This result indicates that the absence of eL39 causes a block in pre-60S r-subunit assembly before rotation of 5S RNP occurs, and before entry of AFs necessary for the export of assembling 60S r-subunits from the nucleoplasm to the cytoplasm.

Next, we compared levels of r-proteins in the Nog2-TAP purified pre-60S r-particles in the presence and absence of eL39. As expected, levels of eL39 decreased most significantly in particles purified from the *rpl39Δ* strain. Importantly, levels of uL4 and uL22, the two other r-protein constituents of the NPET, did not significantly decrease compared to levels of most other r-proteins (Figure 4B). This result indicates that eL39 does not affect loading of uL4 and uL22 into pre-60S r-particles. Thus, defects observed in the absence of eL39 seem to be specific, and are not occurring as a consequence of impaired assembly of the other two r-proteins that associate with the NPET.

### Pre-60S r-particles purified in the absence of eL39 uncover parallel pathways of ribosome assembly

**Figure 5.**
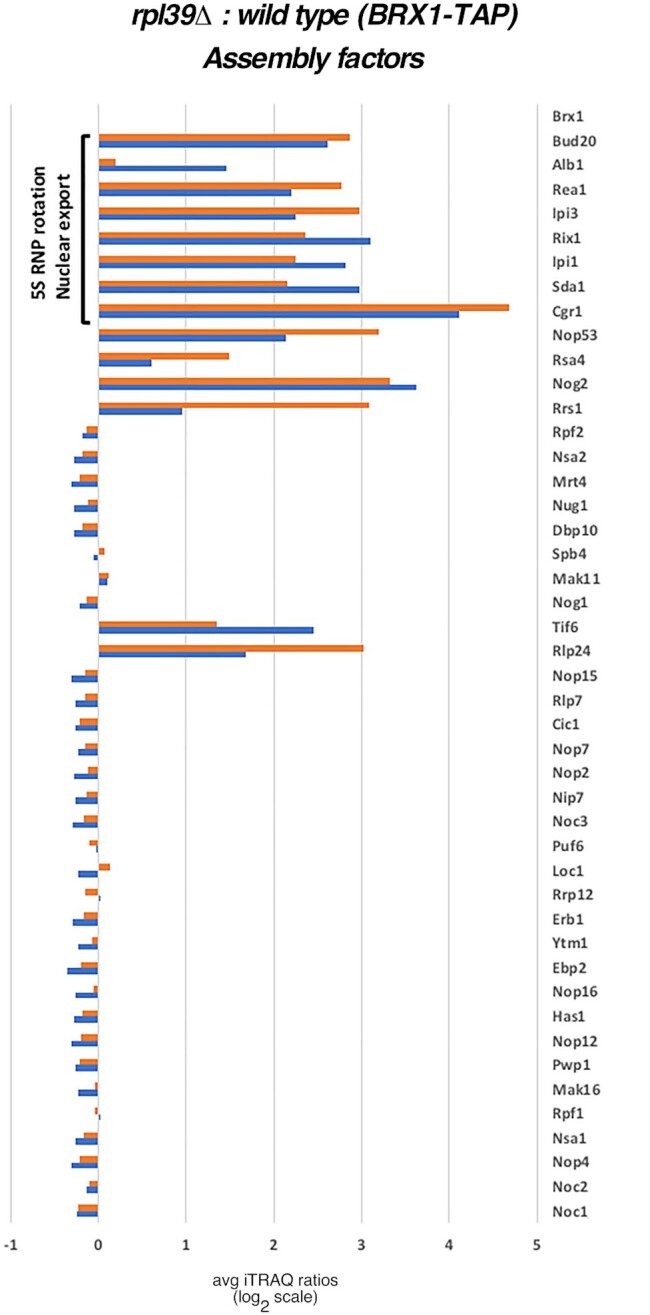
In the absence of eL39, later entering AFs co-purifies with early AF Brx1. Wild-type (JWY8820) and *rpl39Δ* (JWY10646) strains expressing a TAP-tagged version of Brx1 were grown in YEPD at 30°C and shifted to 16°C for 5 h. Assembling 60S r-subunits containing Brx1-TAP were affinity-purified and samples were used for iTRAQ to quantify relative changes in levels of AFs and r-proteins in the *rpl39Δ* mutant compared to the wild-type cells. The ratios were normalized to levels of the bait protein Brx1. The fold-change is shown using bar graphs in log_2_ scale. Blue and red bars represent two biological replicates. AFs involved in 5S RNP rotation and nuclear export of pre-60S r-particles are labelled with a black bracket.

**Figure 6.**
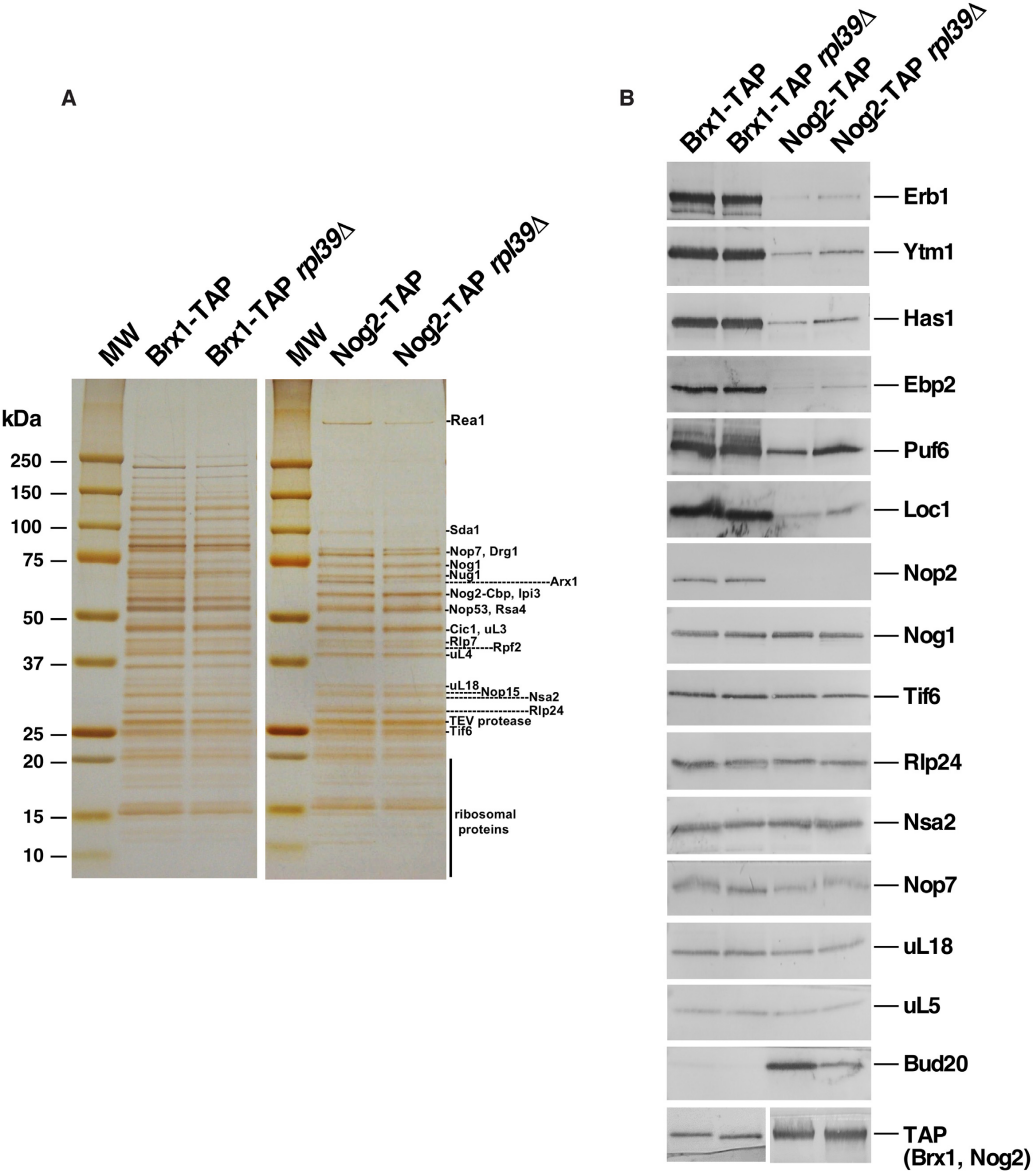
Nog2-containing particles are enriched for a subset of early AFs in cells lacking eL39. Wild-type and *rpl39Δ* strains expressing TAP-tagged versions of Brx1 or Nog2 were grown in YEPD at 30°C and shifted to 16°C for 5 h. Pre-60S r-particles were affinity purified, and their protein constituents were separated by SDS-PAGE and silver-stained (left). Samples were also subjected to western blotting using antibodies against the indicated r-proteins and AFs (right). All samples were derived from the same experiment and all western blot membranes were processed in parallel. The respective bait factors, Brx1-TAP and Nog2-TAP, were used as loading controls.

Surprisingly, in the particles purified *via* Nog2-TAP from *rpl39Δ* cells, we also observed a modest accumulation of a specific subset of early AFs, including Mak16, Ytm1, Erb1, Brx1, Ebp2, Has1, and Puf6. These AFs associate with assembling 60S r-subunits during early nucleolar steps, and dissociate during the transition from state D to E (Mak16), or state E to NE1 (Ytm1, Erb1, Brx1, Ebp2 and Has1). We also observed accumulation of Pwp1 and Nop12, early associating AFs, whose timing of dissociation from pre-60S r-particles remains unknown (Figure 4A and Supplementary Figure S7) (58). Normally, Nog2 enters pre-60S r-subunits after these early AFs exit (during the transition from NE2 to Nog2 state 1 particles) (Supplementary Figure S7) (58,59), and thus it should not co-purify with significant amounts of early AFs. Therefore, to examine in more detail the behavior of these early and late AFs in the *rpl39Δ* mutant, we affinity-purified Brx1-containing pre-ribosomal particles from *RPL39* and *rpl39Δ* cells shifted from 30 to 16°C for 5 h, and examined changes in their protein constituents by iTRAQ (Figure 5). As expected, we did not observe accumulation of any subset of early-associating and early-dissociating AFs in Brx1-containing particles purified from the *rpl39Δ* cells, since these AFs should be present in stoichiometric amounts in early purified particles. However, interestingly, our data showed that levels of normally later-entering AFs were increased. This group included Nog2, AFs involved in 5S RNP rotation (Sda1, Rea1 and members of the Rix1 complex), as well as the export factor Bud20 (Figure 5, see also Supplementary Figure S7). The iTRAQ results were confirmed by SDS-PAGE and western blotting of selected AFs present in purified samples using both Brx1-TAP and Nog2-TAP as baits (Figure 6). Indeed, consistent with the iTRAQ data, western blot analysis showed that Brx1-containing particles lacking eL39 did not accumulate, as expected, early AFs such as Erb1, Ytm1, Has1, Ebp2, Puf6, Loc1 or Nop2. The Nog2 particles showed a small but detectable increase in the levels of these early AFs, confirming the iTRAQ data (Figure 6B). We did not detect any accumulation of Bud20 in Brx1-containing particles, even though it accumulated significantly by iTRAQ, suggesting that the fraction of these particles purified using Brx1-TAP is too low to be detected by western blotting. However, in Nog2-containing particles, as expected, levels of Bud20 decreased.

Taken together, these data suggest that, in the *rpl39Δ* mutant there exists a small subpopulation of particles in which later entering AFs such as Nog2 may load onto pre-60S r-subunits earlier that they normally do, before the early AFs Erb1, Ytm1, Has1, Ebp2, Brx1, Puf6 and Loc1 are released. These results also indicate that eL39 affects more than one step in assembly of 60S r-subunits. The first impairment most likely occurs at state D and/or state E, when these early AFs are normally released, while the second one leads to a delay/block at a step before 5S RNP rotation (Supplementary Figure S7). These results are consistent with the other phenotypes detected upon deletion of *RPL39*: a block in processing of both 27SB and 7S pre-rRNAs and accumulation of pre-60S r-subunits in the nucle(ol)us. Moreover, our results prompted us to speculate that when assembly is blocked or stalled at certain steps, as seems to be the case when eL39 is lacking, potentially less efficient assembly pathways may be favored. Such ‘non-canonical’ particles in which the release of certain early AFs is delayed, were uncovered in a previous investigation (33) as well as in this work. This may reflect the existence of parallel/alternative pathways in 60S r-subunit assembly, as previously observed during assembly of bacterial r-subunits (37–39). Importantly, small amounts of most of the tested early-dissociating AFs were also detected in wild-type Nog2-containing particles, even though they are thought to exit pre-60S particles before Nog2 enters the assembly pathway (Figure 6B). This suggests that parallel/alternative pathways may occur normally even in wild-type conditions, but with much lower efficiency (see Discussion).

To test whether or not these ‘non-canonical’ particles correspond to productive particles and not ‘dead-end’ products that transiently accumulate before being ultimately destined for degradation, we assessed the *in vivo* activity of turnover pathways for pre-60S r-particles in the absence of eL39. To do this, we combined in double mutants the *RPL39* deletion with a mutation partially inactivating the TRAMP complex (e.g. *trf4Δ*) or the exosome (e.g. *rrp6Δ*), and analyzed the levels of 27SB and 7S pre-rRNAs in these conditions. As a result, despite the fact that the *trf4Δ rpl39Δ* and *rrp6Δ rpl39Δ* double mutants are slightly sicker than the individual single mutants (Supplementary Figure S10A), neither 27S nor 7S pre-rRNA species significantly accumulated in these mutants compared to the *rpl39Δ* single mutant strain (Supplementary Figure S10B and C). We conclude that ‘non-canonical’ particles uncovered in this work may be as stable in the presence as in the absence of eL39 and therefore their degradation is not further impaired upon partial inactivation of the turnover machinery, as would be expected if they were mostly ‘dead-end’ intermediates (91).

## DISCUSSION

This work has improved our understanding of the roles of eL39 in both the assembly and function of ribosomes. Consistent with its presence near the exit of the NPET of 60S r-subunits, we found that eL39 is important for proper protein folding of nascent polypeptides, which minimizes subsequent protein aggregation. We suggest that eL39 could be partially loaded onto maturing 60S r-subunits earlier than originally reported, during middle-nucleolar steps of pre-60S r-subunit maturation. In agreement with this, we discovered that eL39 is important for middle-stage, nucleolar assembly of 60S r-subunits, although eL39 is not visualized by cryo-EM in pre-60S r-particles corresponding to those stages. The inability to visualize eL39 in these particles suggests that eL39 could remain in a flexible state, most likely not yet stably accommodated onto the body of these pre-60S r-particles. We found that eL39 is also important for maturation of late nucleolar/early nucleoplasmic r-particles, before 5S RNP rotation, when it completely stabilizes onto pre-60S r-subunits. The delay in 27SB and 7S pre-rRNA processing and the defect in intracellular trafficking of pre-60S particles observed in the absence of eL39 are likely caused by improper NPET maturation, which affects the conformations of rRNA helices H74-76 and H68-H69. These immature or displaced rRNA helices may affect association or dissociation of several AFs. For example, Rea1 is absent from the *rpl39Δ* mutant particles. In turn, this AF is necessary for both the exit of Ytm1 and Erb1 from assembling 60S r-subunits, and for the subsequent rotation of the 5S RNP (92). Similar phenotypes were previously observed in the *rpl4Δ63–87* mutant, defective in uL4, another component of the NPET (33). eL39 fails to assemble into ribosomes in this *rpl4Δ63–87* mutant, perhaps accounting for some of its defects. Surprisingly, we found that a fraction of pre-60S r-particles in the *rpl39Δ* mutant contain both early and late AFs, which are usually not simultaneously associated with assembling r-subunits. This indicates that, in the absence of eL39, the release of a subset of early AFs may be delayed, or later AFs may associate prematurely with pre-60S r-particles. In either case, these particles likely represent alternative/parallel pathways of ribosome assembly (see below), previously observed in the biogenesis of bacterial r-subunits (37–39).

Under laboratory conditions, eL39 is not essential for growth (Supplementary Figure S3). Depletion of most r-proteins, in both large and small r-subunits causes lethality. Nevertheless, about one fifth of all yeast r-proteins are not essential under growth conditions in the laboratory, which are almost always less harsh and restrictive than in nature, where the presence of these highly conserved proteins might confer a strong selective advantage. However, deletion of most of these r-protein genes does in fact cause slow growth even under laboratory conditions (62). In addition to eL39, most other non-essential r-proteins show distinct growth and r-subunit assembly defects (26,27,62,93–101). Conveniently, the non-essential r-proteins enable one to less ambiguously study defects in protein synthesis, since mature ribosomes are still being made in the absence of these proteins, although usually in lower levels than in wild-type cells. Thus, information gathered from deletion of non-essential proteins may in some instances be even more valuable than that from depletion of essential proteins, which do not allow studying steps past the block in assembly, including the proper functioning of ribosomes.

It has been shown previously that ribosomes lacking eL39 display hypersensitivity to paromomycin (25). This drug reduces translational accuracy by increasing the affinity of the A-site for tRNA (102). Thus, it has been suggested that the absence of eL39 allows ribosomes to translate faster but with a four-fold increase in error frequency (25). These results raise a question: How is the effect of the absence of eL39 communicated to the PTC in the large r-subunit and to the A-site in the small r-subunit? eL39 is localized in the NPET, distant from the tRNA-binding A-site in the small r-subunit. It has been proposed that interactions of the nascent polypeptide chain with the tunnel might be relevant to functionally connect the PTC to the NPET exit (32,103). Since the absence of L39 makes translation faster but error-prone, it is likely that eL39 could serve as part of a barrier to the nascent chain and ensures that translation does not occur at an unsafe speed. This barrier is made up of interactions between positively charged residues of eL39 (highly exposed in the NPET), nascent polypeptide chains, and the tip of rRNA helix H24, which is a flexible tetraloop positioned at the NPET exit (32).

Our data also suggest that the absence of eL39 may also affect translational fidelity directly by hindering proper maturation of the PTC during 60S r-subunit assembly. Cryo-EM of *rpl4Δ63–87* mutant pre-ribosomes revealed that failure of the NPET to mature affects proper folding of several helices, including helices H68 and H69 in the PTC. Since the absence of eL39 causes a block at the same step of 60S r-subunit biogenesis as the uL4Δ63–87 truncated r-protein, it is possible that maturation of helices H68 and H69 is also compromised in the *rpl39Δ* mutant, such that mature 60S r-subunits can form but have mild defects in protein synthesis. This is further supported by the observation that the absence of eL39 causes cold sensitivity. If eL39 is important for proper folding of pre-rRNA, e.g. helices H68 and H69, pre-ribosomes lacking eL39 may not be able to continue proper maturation at low temperatures. Under these circumstances, rRNA may be kinetically trapped in unproductive conformations, as a consequence of the lack of eL39. In contrast, at higher temperatures, rRNA may be able to escape such traps and fold properly, driving more maturing particles toward completion of ribosome assembly (104,105). Indeed, growth of the *rpl39Δ* strain is less affected as the temperature rises, and the growth defect is undetectable at 37°C, although a ribosome biogenesis defect still persists at this temperature (Figure 2 and Supplementary Figure S3).

In mature 60S r-subunits, eL39 binds to domain III of 25S rRNA. This domain is not stabilized until the assembly of pre-60S r-subunits reaches the so-called state D intermediate stages (Supplementary Figure S7). The resolution of the cryo-EM model of state D is 4.3 Å, which makes visualization of any potential density for eL39 challenging. However, the 3.3 Å resolution of state E particles allows visualization of limited eL39 densities (Supplementary Figure S8), which is consistent with the possibility that eL39 enters nascent ribosomes earlier than previously thought (Figure 3). The same limited densities can be visualized in states NE1 and NE2 (resolution 3.9 and 3.8 Å, respectively) (data not shown). Thus, our data imply that eL39 fully stabilizes into the NPET only after state NE2, but it associates with state C particles (or earlier) in an incompletely accommodated state. Consistent with these observations, our results also strongly suggest that eL39 affects pre-60S r-subunit assembly at states D/E, when it is still not completely accommodated into particles.

**Figure 7.**
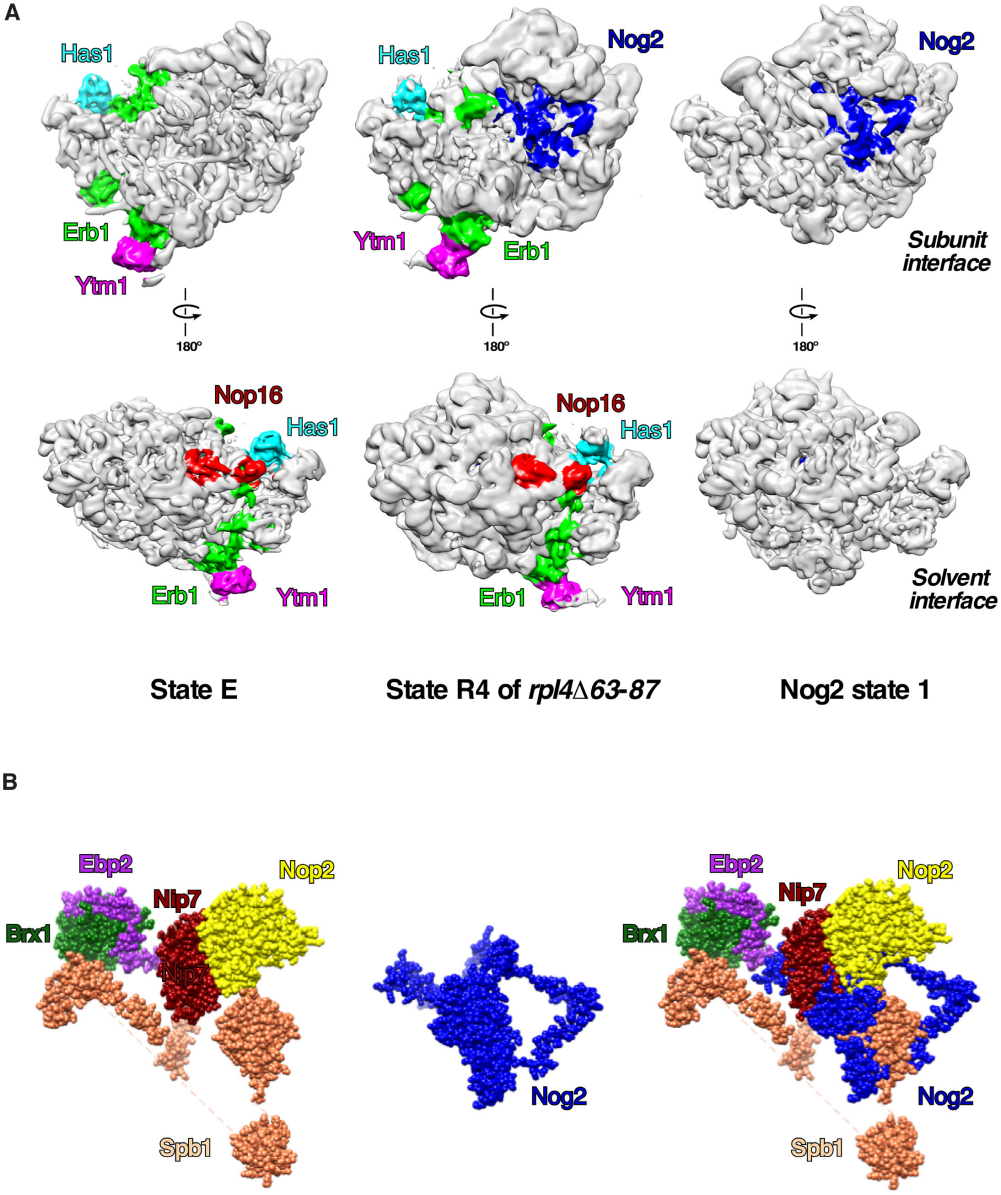
Only a subset of early AFs can stay bound to pre-60S r-subunits after association of Nog2, while others sterically clash with Nog2. (**A**) Density maps for the cryo-EM structures of pre-60S state E, R4 from the *rpl4Δ63–87* mutant and Nog2 state 1 particles are shown. Extra densities correspond to early AFs Ytm1 (magenta), Erb1 (green), Has1 (cyan), and Nop16 (red). Nog2 is colored in blue. Densities are colored using the color zone tool from Chimera. The central protuberance containing 5S RNP is still not visible in state E particles, but is present in R4 and Nog2 state 1 particles. (**B**) Structure of Nog2 from the Nog2 state 1 particle (middle) was superimposed to structures of Brx1, Ebp2, Nop2, Nip7 and Spb1 from state E particles (left). Note that Nop2, Nip7 and Spb1 sterically clash with Nog2 binding in the same pre-60S precursor, while Brx1 and Ebp2 do not (right).

Previous studies with *E. coli* suggest that ribosome assembly occurs *via* multiple parallel pathways rather than through a single rate-limiting step. The entire process is dynamic and can be ‘re-routed’ through different pathways as needed (37,39). This raises an important question: do alternative pathways in assembly of r-subunits also exist in eukaryotes? Hints that parallel pathways might exist in wild-type conditions in yeast have been reported, evident by co-purification with Nog2 of small amounts of AFs that are thought to disassociate from pre-60S r-subunits by states E or NE1, before Nog2 assembles (79,86) (see Supplementary Figure S7). Alternative pathways have also been previously reported for the timing of the removal of the internal transcribed spacer 2 (ITS2) relative to rotation of the 5S RNP and maturation of the PTC (86,106). For example, using cryo-EM, we have visualized intermediates in mutants blocked in steps before 5S RNP rotation. Some of those particles contained ITS2 and its associated AFs, and in others ITS2 was fully processed (33,107). Here, we report another example of alternative/parallel pathways in the assembly of the pre-60S r-subunits. Our cryo-EM data with pre-60S r-subunits from the *rpl4Δ63–87* mutant uncovered non-canonical, so-called R4 particles, resembling Nog2 state 1, but containing extra densities for early AFs Ytm1, Erb1, Has1 and Nop16, which are thought to exit pre-60S r-particles before Nog2 enters (33). In these R4 particles, the 5S RNP is visible but not yet rotated, ITS2 is present, and densities for Arx1, Bud20 and Nop53 are missing ((33); see also Figure 7). These cryo-EM data for the *rpl4Δ63–87* mutant particles are in agreement with the biochemical data obtained for the *rpl39Δ* mutant. As previously proposed (11), the coordinated removal of Erb1, Ytm1, Nop16, and Has1 is required for binding of Nop53 to the Nog2 state 1 particle and subsequent ITS2 processing. Thus, the retention of ITS2 in these ‘chimeric’ R4 particles is consistent with the presence of these four early AFs and the absence of Nop53. Erb1 was proposed to act as a central coordinator of remodeling events at the inter-subunit side of the pre-60S r-particles in states D and E, by influencing the release of Has1, Nop16, Spb1, Noc3, Ebp2 and Brx1 (10). Although this is very likely the case in fast growing wild-type cells, our data suggest that, when the assembly pathway is stalled or blocked, some steps may occur ‘out of order’, and some of these AFs may be able to dissociate independently from Erb1. Thus, AFs such as Spb1, Nop2 and Nip7, which sterically clash with the binding site for Nog2 (see Figure 7B), may exit assembling particles independently from Erb1, allowing Nog2 to load earlier into pre-60S particles. Early AFs Ytm1, Erb1, Has1 and Nop16 that are visualized in the R4 particles from the *rpl4Δ63–87* mutant are located on the periphery of assembling particles (see Figure 7), and therefore should not hinder loading of Nog2 and the 5S RNP. However, RNA helices H68-H69 of domain IV, and H74-H79 of domain V are not stabilized onto the pre-60S r-subunit in R4 particles (33). Thus, the exit of Ytm1, Erb1, Has1 and Nop16 may be needed for rearrangement of these helices, as previously suggested (10).

**Figure 8.**
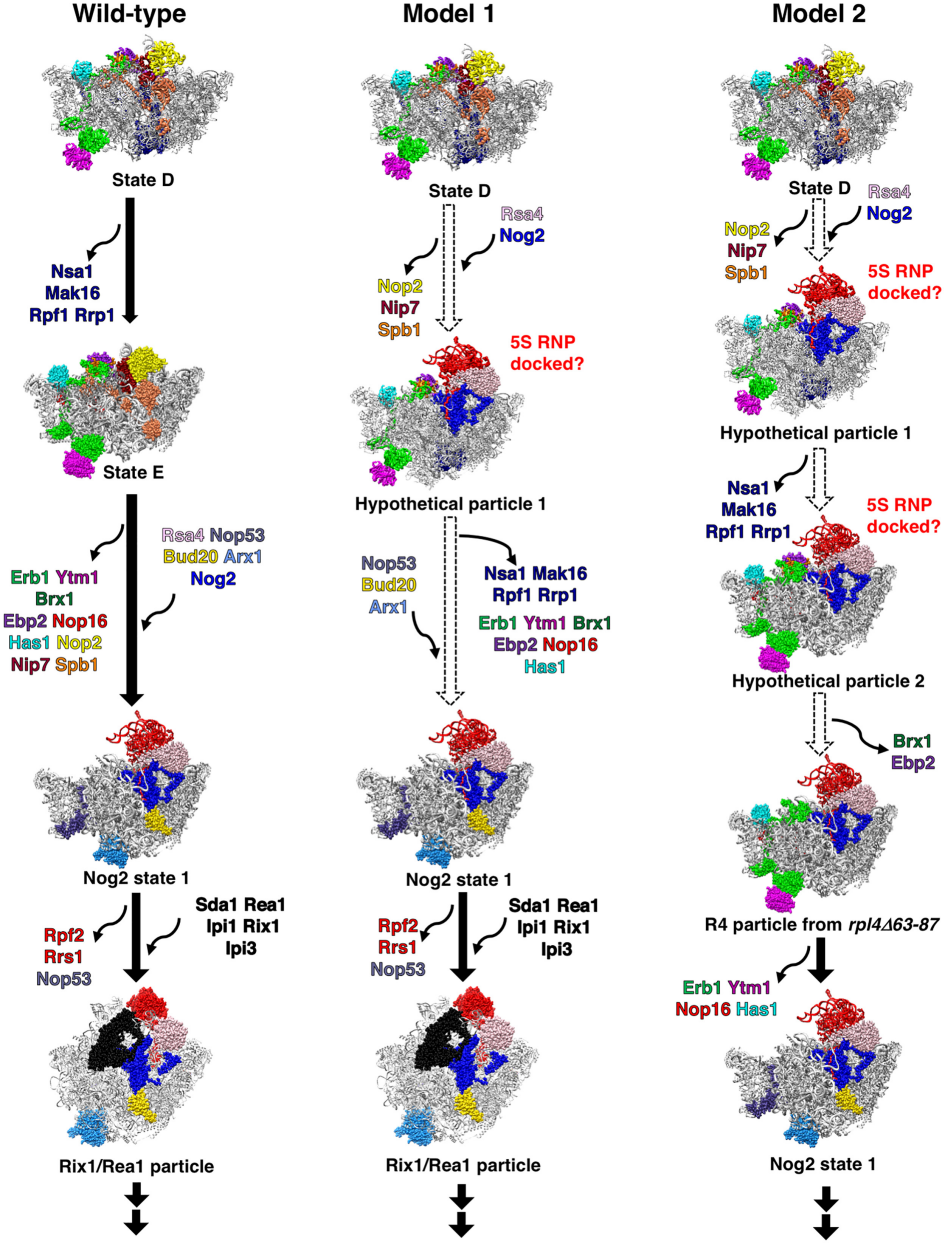
Hypothetical alternative pathways during assembly of the yeast 60S r-subunit. The left panel represents the standard model for the 60S r-subunit assembly pathway in wild-type cells, while the middle and right panels represent two different models for potential alternative pathways. The thick black arrows represent the sequential order of assembly under wild-type conditions. Dashed white arrows represent hypothetical alternative pathways based on the iTRAQ data from the *rpl39Δ* mutant. Models for early AFs from atomic models of states D and E were superimposed onto atomic models of Nog2 state 1 or Rix1/Rea1 particles. Particles NE1 and NE2 were omitted for simplification. All hypothetical structures were labeled as such. RNA, and non-relevant r-proteins and AFs are colored grey. For simplicity, helices H68-H69 and H75-H79 were included in the R4 particle, although their densities are missing from the cryo-EM density map of R4 particle. Additionally, potential conformational changes in RNA structures in any of the hypothetical particles were not presented in this model. PDB IDs 6EM5, 6ELZ, 3JCT, and 5FL8 were used for this figure.

Our results on the *rpl39Δ* mutant suggest the existence of other non-canonical particles in addition to the R4 particle discussed above. Based on these results, we speculate that there are two potential models of minor and alternative pathways for 60S r-subunit assembly. Model 1 takes into account all early AFs that increase in the absence of eL39 in Nog2-TAP containing particles. We assume that are all in the same particle named ‘hypothetical particle 1’ (Figure 8, middle panel). This particle contains the Nsa1 module and factors such as Ebp2, Brx1, Ytm1, Erb1, Has1 and Nop16, together with Nog2. The presence of the Nsa1 module is possible as it is located on the solvent-exposed surface of this particle while that of Ytm1, Erb1, Has1 and Nop16 is possible as they are located on its periphery. Thus, none of these factors are likely to affect the loading of Nog2 or anchoring of the 5S RNP complex. In agreement with this scenario, it has been reported that Nsa1 can remain associated with pre-60S particles travelling to the cytoplasm in case of its inefficient release upon mutational inactivation of Rix7 (108). The presence of Ebp2 and Brx1 on these particles may also be tolerated, as no steric hindrance is likely expected between them and the non-rotated 5S RNP complex. Exit of all these early AFs may allow maturation to the Nog2 state 1 particle. Model 2 takes into consideration the R4 particle previously reported (33), and includes two hypothetical particles (Figure 8, right panel): ‘hypothetical particle 1’, which is identical to the previously discussed particle, and ‘hypothetical particle 2’ where the Nsa1 module has dissociated but Brx1 and Ebp2 have not. The further release of these two factors would give rise to the R4 particle, which as above mentioned still contains Ytm1, Erb1, Has1 and Nop16. As for model 1, the release of these AFs allows formation of the Nog2 state 1 particle.

We believe that when ribosomal particles are blocked in certain steps of maturation, AFs with the potential to prematurely bind to the particles may drive reactions to circumvent the block, by following alternative maturation pathways. We favor a model where the absence of eL39 increases the likelihood of a switch to alternative pathways due to its long-range effects on the conformations of H68-69, as previously reported for the *rpl4Δ63–87* mutant (33). Since Nog2 and the C-terminal extension of Rpf2 are both in close contact with H69, it is possible that premature entry of Nog2 and accommodation of Rpf2 (together with the 5S RNP and Rrs1) may help overcome inefficient maturation of H68-H69, increasing the possibilities of using an alternative, less ‘efficient’ maturation pathway. In this context, the absence of the Rpf2 C-terminal extension in the *rpf2Δ255–344* mutant may explain why alternative pathways seem not to increase in that strain, as inferred from our iTRAQ analysis (Supplementary Figure S11). Thus, even in the presence of Nog2, the lack of the C-terminal extension of Rpf2 would prevent stabilization of H68-H69 (107).

In general, what is the advantage/biological significance of parallel pathways? The biogenesis of r-subunits is a series of simultaneous, semi-autonomous but interconnected events. When the more efficient wild-type pathway is delayed/stalled (e.g. by the absence of eL39), yeast cells might switch to potentially less ‘efficient’ alternative pathways; this scenario is similar to that which occurs for bacterial 50S r-subunit assembly in the absence of bL17 (38). In yeast, this re-routing of 60S r-subunit assembly through alternative pathways is likely not specific to the absence of eL39; copurification of early AFs such as Drs1, Erb1 and Has1 with Nog2 has been observed upon depletion of uL2, uL23 or eL43 (79). Even in wild-type cells, parallel pathways may exist to allow for more flexible r-subunit assembly. Thus, parallel pathways can give cells a selective advantage for survival by driving the ribosome biogenesis pathway forward when otherwise assembling r-subunits would be stalled and potentially turned over by the surveillance machinery. Further studies are clearly required to understand in which instances usage of alternative pathways increases, what causes the shift to parallel pathways, and their exact significance in r-subunit assembly.

## DATA AVAILABILITY

The data that support this work are available from the corresponding authors upon reasonable request.

## Supplementary Material

gkac366_Supplemental_File
