# Supplementary material for: Ribosomal protein eL39 is important for maturation of the nascent polypeptide exit tunnel and proper protein folding during translation
Source: Nucleic Acids Res. 2022 May 27;50(11):6453–73. doi: 10.1093/nar/gkac366 (PMC9226512; doi:10.1093/nar/gkac366)
Supplement: gkac366_Supplemental_File [file gkac366_supplemental_file.pdf]

SUPPLEMENTARY MATERIAL TO THE MANUSCRIPT

Ribosomal protein eL39 is important for maturation of the nascent polypeptide exit tunnel and proper protein folding during translation

Jelena Micic[1,6], Olga Rodríguez-Galán[2,3,6], Reyes Babiano[2,3], Fiona Fitzgerald[1], José Fernández-Fernández[2,3], Yunyang Zhang[4], Ning Gao[4], John L. Woolford Jr.[1,5], and Jesús de la Cruz[2,3,5]

[1] Department of Biological Sciences, Carnegie Mellon University, Pittsburgh, PA, USA.

[2] Instituto de Biomedicina de Sevilla, Hospital Universitario Virgen del Rocío/CSIC/Universidad de Sevilla, Seville, Spain.

[3] Departamento de Genética, Universidad de Sevilla, Seville, Spain.

[4] State Key Laboratory of Membrane Biology, Peking-Tsinghua Joint Centre for Life Sciences, School of Life Sciences, Peking University, Beijing, China.

[5] To whom correspondence should be addressed.

[6] These authors contributed equally to this work as first authors.

SUPPLEMENTARY TABLES

Supplementary Table S1. Yeast strains used in this study

Strain	Relevant genotype	Source
W303-1A	*MAT***a** *ade2-1 his3-11,15 leu2-3,112 trp1-1 ura3-1*	(1)
BY4741	*MAT***a** *his3Δ1 leu2Δ0 lys2Δ0 ura3Δ0*	Euroscarf
BY4742	*MATα his3Δ1 leu2Δ0 lys2Δ0 ura3Δ0*	Euroscarf
ORY211	As W303-1A but *rpl39::*natNT2	This work
ORY153	As BY4742 but *rpl39::*kanMX4	(2)
JDY532	As W303-1A but *ssb1::*HIS3MX6 *ssb2::*natNT2	(3)
JDY509	As W303-1A but *zuo1*::HIS3MX6	This work
JDY532	As W303-1A with *ssb1*::HIS3MX6 *ssb2*::natNT2	(3)
JWY11983	*MAT***a** *his3-Δ200 leu2-Δ1 lys2-801 trp1-Δ101 ura3-52 nog2::NOG2-TAP (URA3)*	This work
JWY11985	As JWY11983 but *rpl39::*kanMX4	This work
JWY8820	As BY4741 but *brx1::BRX1-TAP* (HIS3MX6)	Horizon
JWY10646	As JWY8820 but *rpl39::*kanMX4	This work
JWY8809	As BY4741 but *nog2::NOG2-TAP* (HIS3MX6)	Horizon
JWY8812	As BY4741 but *ssf1::SSF1-TAP* (HIS3MX6)	Horizon
JWY11694	As JWY8809 but *rpl39::RPL39-13MYC* (kanMX4)	This work
JWY11692	As JWY8812 but *rpl39::RPL39-13MYC* (kanMX4)	This work
ORY77	As W303-1A but *trf4::*HIS3MX4	Gift from Dr. D. Kressler
ORY149	As W303-1A but *rrp6::*kanMX4	Gift from Dr. A. Morillon (YAM587)
JDY1316	As W303-1A but *rpl39*::natNT2 *trf4::*HIS3MX4	This work
JDY1317	As W303-1A but *rpl39*::natNT2 *rrp6::*kanMX4	This work

Supplementary Table S2. Plasmids used in this study

Name	Relevant information	Source
YCplac33	*CEN, URA3*	(4)
YCplac111	*CEN, LEU2*	(4)
YCplac33-RPL39	eL39*; CEN, URA3*	This work
YCplac111-RPL39	eL39; *CEN, LEU2*	This work
YCplac111-yEGFP	C-terminal yEGFP; *CEN, LEU2*	Hall lab
YCplac-111-RPL39-yEGFP	eL39-yEGFP; *CEN, LEU2*	This work
pRS316-RPL25-yEGFP-mRFP-NOP1	uL23-yEGFP mRFP-Nop1; *CEN, URA3*	(5)
pRS316-RPS3-yEGFP-mRFP-NOP1	uS3-yEGFP mRFP-Nop1; *CEN, URA3*	(5)
pRS315-RPL25-yEGFP-mRFP-NOP1	uL23-yEGFP mRFP-Nop1; *CEN, LEU2*	(5)
pRS314-NOP1mRFP	mRFP-Nop1; *CEN, TRP1*	(5)
pRS316-*GAL-NMD3FL*	*GAL::NMD3, CEN, URA3*	(6)
pRS316-GAL-NMD3Δ100	*GAL::NMD3Δ100, CEN, URA3*	(6)
pAG25	*pFA6, natMX4*	(7)
pBS1539	*TAP-tag URA3 marker*	(8)
pAF6a-13Myc-KanMX6	*C-terminal tagging of eL39*	(9)

Supplementary Table S3. Oligonucleotides used in this study

Name	5'-3' Sequence
L39-up	GCAGAGGTGGTCAAGATGACG
L39-dw	GGAATGGCTACTCATTTACCG
L39Nat-up	AATTCGAAAAAGACAAGCAAATAAACACAGATAGATCAACCAGCTGAAGCTTCGTACGCT
L39Nat-low	ACAAATGACAAAAAGTTTGAAGCATAAATATGTTCTTCGCCTAGTGGATCTGATATCATC
EcoRI-L39-up	GAATTCCCACACTTGTTCAATCTACC
L39-XbaI-dw	TCTAGAGATGTTCATCTTGGTTCTTC
Probe b (18S)	CATGGCTTAATCTTTGAGAC
Probe c (3-D/A_2)	GACTCTCCATCTCTTGTCTTCTTG
Probe d (A_2/A_3)	TGTTACCTCTGGGCCC
Probe e (5.8S)	TTTCGCTGCGTTCTTCATC
Probe f (E/C_2)	GGCCAGCAATTTCAAGTTA
Probe g (C_1/C_2)	GAACATTGTTCGCCTAGA
Probe h (25S)	CTCCGCTTATTGATATGC
Probe 5S	GGTCACCCACTACACTACTCGG
Probe U2	GAACAGATACTACACTTGATCTAAGC
L39-MYC-UP	TATCCGTTACAACGCTAAGAGAAGAAACTGGAGAAGAACCCGGATCCCCGGGTTAATTAA
L39-MYC-DN	AAGTTTGAAGCATAAATATGTTCTTCGCTTAGATGTTCATGAATTCGAGCTTCGTTAAAC

LEGENDS TO THE SUPPLEMENTARY FIGURES

Supplementary Figure S1. Pre-rRNA processing in *S. cerevisiae*. (**A**) Structure of an rDNA repeat unit. Each unit contains two independently transcribed elements. The long element is transcribed by RNA polymerase I (RNAP I) into a polycistronic pre-rRNA encoding the 18S, 5.8S and 25S rRNAs. The short element is transcribed by RNA polymerase III (RNAP III) into a pre-5S rRNA. Non-transcribed, external and internal transcribed spacers (NTSs, ETSs and ITSs) are indicated. The mature rRNA species are shown as bars and the spacers as lines. The transcription start sites are highlighted by red arrows. The processing sites and the location of the hybridization probes, listed in Table S3, are shown. (**B**) Scheme of the pre-rRNA processing pathway. The RNAP I transcript can undergo either co- or post-transcriptional processing, leading to generation of the 20S and 27SA$_2$ pre-rRNAs, which are components of early pre-40S and pre-60S ribosomal particles, respectively. These pre-rRNAs are further processed into the mature 18S and 5.8S and 25S rRNAs by specific cleavage and trimming reactions. This figure has been adapted from that shown in García-Gómez *et al.* (10). For further description of the yeast pre-rRNA processing pathway, see (11,12).

Supplementary Figure S2. The nascent polypeptide exit tunnel is comprised of rRNA and three r-proteins uL4, uL22, and eL39. (A) Internal loops of uL4 (cyan) and uL22 (orange) form the constriction sites of the NPET. **(B)** The presence of eL39 (green) in the NPET reduces the radius of the tunnel. Shown in grey are the portions of rRNA domains I, II, III, IV, and V that comprise the rRNA forming the NPET. PDB ID 3JCT (Nog2 state 1; (13)) was used for this figure.

Supplementary Figure S3. The *rpl39Δ* mutant exhibits a slow growth phenotype, enhanced at low temperatures. (**A**) Whole cell extracts were prepared from BY4742 (*Wild type*) and ORY153 (*rpl39Δ*) cells grown in YEPD at 30°C. Equivalent amounts of cell extracts were separated by SDS/PAGE and levels of the eL39 and uL1 r-proteins were determined by western blot analysis using specific antibodies. (**B**) Isogenic BY4742 (*Wild type*) and ORY153 (*rpl39Δ*) strains, were grown in liquid YEPD at 30°C and diluted to an OD_{600} of 0.05. Serial dilutions were spotted onto YEPD medium, and incubated at 26, 30 and 37°C for 3 days or at 16 and 22°C for 6 days. (**C**) Similar results were obtained in the W303 background. Isogenic W303-1A (*Wild type*) and ORY211 (*rpl39Δ)* strains were spotted in 10-fold serial dilutions onto YEPD medium and incubated at 26, 30 and 37°C for 3 days, or at 22°C for 6 days.

Supplementary Figure S4. eL39 is required for biogenesis of 60S r-subunits. (**A**) Yeast strains BY4742 (*Wild type*) and ORY211 (*rpl39Δ*) were grown in YEPD medium at 22, 30 and 37°C to an OD_{600} of around 0.8. Cells were harvested and total cell extracts were prepared. Ten A_{260} units of each extract were resolved on 7 to 50% sucrose gradients, and assayed by continuous monitoring of A_{254}. Sedimentation is from left to right. The peaks of free 40S and 60S r-

subunits, 80S free couples/monosomes, and polysomes are indicated. Half-mer polysomes are labelled by arrows. (**B**) Quantification of the abundance of 60S r-subunits relative to 40S subunits. The above strains were grown in YEPD medium at 22 or 30°C to an OD_{600} of around 0.8. Cell extracts were prepared under polysome run-off conditions, by the omission of cycloheximide, in a buffer lacking $MgCl_2$ to dissociate ribosomes into free 40S and 60S r-subunits. Ten A_{260} units of each extract were resolved in 7-50% sucrose gradients prepared in the same buffer. The A_{254} was continuously measured. Sedimentation is from left to right. The peaks of total 40S and 60S r-subunits are indicated. The 60S/40S ratio for the *rpl39Δ* strain was calculated and normalized to that for the wild-type control, which was set at 1.0 at each temperature. Data are the average of three independent experiments; the relative standard deviation was less than 5% in each case.

Supplementary Figure S5. Wild-type and GFP-tagged eL39 complement the *rpl39Δ* mutant. (**A**) The strain ORY153 (*rpl39Δ)* was transformed with YCplac111 (+ vector), YCplac111-RPL39 (+ *RPL39*) or YCplac111-RPL39-yEGFP (+ *RPL39-yEGFP*), grown in liquid SD-Leu medium and diluted to an OD_{600} of 0.05. A 10-fold series of dilutions were plated in SD-Leu medium, and incubated at 30°C and 37°C for 3 days, and at 22°C for 6 days. (**B**) The above strains were grown in liquid SD-Leu medium at 30°C to an OD_{600} of around 0.8. Cells were harvested and total cell extracts were prepared. Ten A_{260} units of each extract were resolved on 7-50% sucrose gradients. The A_{254} was continuously monitored. Sedimentation is from left to right. The peaks of free 40S and 60S r-subunits, 80S free couples/monosomes, and polysomes are indicated. Half-mer polysomes are labeled by arrows. Note that expression of eL39-yEGFP does not totally complement the polysome defect of the *rpl39Δ* mutant.

Supplementary Figure S6. The absence of eL39 leads to nuclear retention of pre-60S r-subunits, which is exaggerated in the cold. BY4742 (*Wild type*) and ORY153 (*rpl39Δ*) strains were transformed with a plasmid that co-expressed mRFP-Nop1 and uL23-yEGFP from their cognate promoters. Selected transformants were grown in liquid SD-Leu medium at 30°C to an OD_{600} of around 0.8 or shifted to 13°C for 8 h. The subcellular localization of the GFP-tagged uL23 r-protein and the mRFP-Nop1 nucleolar marker was analyzed by fluorescence microscopy. Merged images reveal accumulation of maturing pre-60S r-particles in the nucleolus in the *rpl39Δ* mutant. The selected cells are representative of approximately 200 cells from each experiment. Scale bar, 5 µm.

Supplementary Figure S7. Simplified pre-60S r-subunit assembly pathway. Atomic models used are: 6EM3, 6EM4, 6EM1, 6EM5, 6ELZ (14), 6YLX, 6YLY (15), 3JCT (13), 5FL8 (16), and 5H4P (17). The timing of dissociation of Pwp1 and Nop12 from the assembly pathway is unknown. Only relevant AFs are marked. The r-proteins are omitted for simplification. Brx1

and Nog2 used as baits for purifications are colored green and purple, respectively. This figure has been adapted from one in Klinge and Woolford (18).

Supplementary Figure S8. Only partial densities for eL39 are visualized in the state E particles. The atomic model for eL39 from the Nog2 state 1 model was fitted into the density maps of state E particles (top), or Nog2 state 1 particles (bottom, used as a control for the presence of eL39). Red color indicates the portion of eL39 for which densities are present, and cyan indicates lack of eL39 density. Cryo-EM density maps for state E and Nog2 state 1 used are EMD-3891 (14) and EMD-6615 (13), respectively.

Supplementary Figure S9. eL39 protein assembles in the nucleolus. Localization of GFP-tagged uL23 and eL39 r-proteins upon induction of the dominant-negative *NMD3Δ100* allele. Cells of the W303-1A strain expressing Nop1-mRFP and uL23-yEGFP from plasmid pRS315-RPL25-yEGFP-mRFP-NOP1 and harboring an empty pRS314 plasmid and cells of the ORY211 strain expressing Nop1-mRFP and eL39-yEGFP from plasmids pRS314-NOP1-mRFP and YCplac111-RPL39-yEGFP, respectively, were transformed with the pRS316-GAL-NMD3Δ100 plasmid. Transformants were grown in liquid SRaf-Leu-Ura-Trp medium at 30°C or shifted to liquid SGal-Leu-Ura-Trp medium at 30°C for 6 h to fully induce the dominant-negative Nmd3Δ100 protein. The GFP and RFP signals were visualized by fluorescence microscopy. Arrows indicate the position of the nucleolus in selected cells.

Supplementary Figure S10. Accumulation of 27S and 7S pre-rRNAs in the absence of eL39 is not dependent on the presence of the TRAMP component Trf4 and the nuclear exosome component Rrp6. (**A**) Strains ORY77 (*trf4Δ*) and ORY149 (*rrp6Δ*) were crossed to a MATα derivative of ORY211 (*rpl39Δ*) and tetratypes were selected. Two representative tetratypes were grown in liquid YEPD at 30°C and diluted to an OD_{600} of 0.05. Then, serial dilutions were spotted onto YEPD medium, and incubated at 30°C for 3 days. Note that both *trf4Δ and rrp6Δ* mutations slightly enhanced the growth defect of the *rpl39Δ* mutant. (**B**) The same strains were grown in liquid YEPD at 30°C, shifted for 5 h at 16°C and harvested. Then, total RNA was extracted and equal amounts of total RNA were subjected to northern hybridization. All bands were detected in the same gel. Probes, between parentheses are described in Supplementary Table S3. (**C**) Quantification of the hybridization signals for the 27S (top) and 7S (bottom) pre-rRNAs. Signal intensity was measured by phosphorimager scanning; values were first normalized to levels of U2 snRNA and, then, to the value obtained for the wild-type control which was arbitrarily set at 1.0. Each value represents the mean from three independent experiments, and the error bars the standard deviations. The arbitrary Significance levels were determined by Student's *t* test (*, $P < 0.05$).

Supplementary Figure S11. Nog2-associated particles do not accumulate early assembling/early dissociating AFs in cells expressing *rpf2Δ255-344*. Wild-type and *rpf2Δ255-344* strains were grown at 30°C and shifted to 16°C for 5 h. Nog2-containing r-particles were purified, and samples were used for iTRAQ to quantify relative changes in levels of AFs. The ratios were normalized to levels of the bait protein (Nog2). The fold-change is shown using bar graphs in $\log_2$ scale. Blue and red bars represent two biological replicates. AFs that disassociate from the assembly pathway during early stages of assembly, and AFs involved in 5S RNP rotation and nuclear exit are labelled with black brackets.

REFERENCES

1. Thomas, B.J. and Rothstein, R. (1989) Elevated recombination rates in transcriptionally active DNA. *Cell*, **56**, 619-630.
2. Steffen, K.K., McCormick, M.A., Pham, K.M., Mackay, V.L., Delaney, J.R., Murakami, C.J., Kaeberlein, M. and Kennedy, B.K. (2012) Ribosome deficiency protects against ER stress in *Saccharomyces cerevisiae*. *Genetics*, **191**, 107-118.
3. Martín-Villanueva, S., Fernández-Pevida, A., Kressler, D. and de la Cruz, J. (2019) The Ubiquitin moiety of Ubi1 is required for productive expression of ribosomal protein eL40 in *Saccharomyces cerevisiae*. *Cells*, **8**, e850.
4. Gietz, R.D. and Sugino, A. (1988) New yeast-*Escherichia coli* shuttle vectors constructed with *in vitro* mutagenized yeast genes lacking six-base pair restriction sites. *Gene*, **74**, 527-534.
5. Ulbrich, C., Diepholz, M., Bassler, J., Kressler, D., Pertschy, B., Galani, K., Böttcher, B. and Hurt, E. (2009) Mechanochemical removal of ribosome biogenesis factors from nascent 60S ribosomal subunits. *Cell*, **138**, 911-922.
6. Belk, J.P., He, F. and Jacobson, A. (1999) Overexpression of truncated Nmd3p inhibits protein synthesis in yeast. *RNA*, **5**, 1055-1070.
7. Goldstein, A.L. and McCusker, J.H. (1999) Three new dominant drug resistance cassettes for gene disruption in *Saccharomyces cerevisiae*. *Yeast*, **15**, 1541-1553.
8. Rigaut, G., Shevchenko, A., Rutz, B., Wilm, M., Mann, M. and Séraphin, B. (1999) A generic protein purification method for protein complex characterization and proteome exploration. *Nat. Biotechnol.*, **17**, 1030-1032.
9. Longtine, M.S., McKenzie III, A., Demarini, D.J., Shah, N.G., Wach, A., Brachat, A., Philippsen, P. and Pringle, J.R. (1998) Additional modules for versatile and economical PCR-based gene deletion and modification in *Saccharomyces cerevisiae*. *Yeast*, **14**, 953-961.
10. García-Gómez, J.J., Fernández-Pevida, A., Lebaron, S., Rosado, I.V., Tollervey, D., Kressler, D. and de la Cruz, J. (2014) Final pre-40S maturation depends on the functional integrity of the 60S subunit ribosomal protein L3. *PLoS Genet.*, **10**, e1004205.
11. Fernández-Pevida, A., Kressler, D. and de la Cruz, J. (2015) Processing of preribosomal RNA in *Saccharomyces cerevisiae*. *Wiley Interdiscip. Rev. RNA*, **6**, 191-209.
12. Henras, A.K., Plisson-Chastang, C., O'Donohue, M.F., Chakraborty, A. and Gleizes, P.E. (2015) An overview of pre-ribosomal RNA processing in eukaryotes. *Wiley Interdiscip. Rev. RNA*, **6**, 225-242.
13. Wu, S., Tutuncuoglu, B., Yan, K., Brown, H., Zhang, Y., Tan, D., Gamalinda, M., Yuan, Y., Li, Z., Jakovljevic, J. *et al.* (2016) Diverse roles of assembly factors revealed by structures of late nuclear pre-60S ribosomes. *Nature*, **534**, 133-137.
14. Kater, L., Thoms, M., Barrio-García, C., Cheng, J., Ismail, S., Ahmed, Y.L., Bange, G., Kressler, D., Berninghausen, O., Sinning, I. *et al.* (2017) Visualizing the assembly pathway of nucleolar pre-60S ribosomes. *Cell*, **171**, 1599-1610 e1514.

15. Kater, L., Mitterer, V., Thoms, M., Cheng, J., Berninghausen, O., Beckmann, R. and Hurt, E. (2020) Construction of the central protuberance and L1 stalk during 60S subunit biogenesis. *Mol. Cell*, **79**, 615-628.e615.
16. Barrio-García, C., Thoms, M., Flemming, D., Kater, L., Berninghausen, O., Bassler, J., Beckmann, R. and Hurt, E. (2016) Architecture of the Rix1-Rea1 checkpoint machinery during pre-60S-ribosome remodeling. *Nat. Struct. Mol. Biol.*, **23**, 37-44.
17. Ma, C., Wu, S., Li, N., Chen, Y., Yan, K., Li, Z., Zheng, L., Lei, J., Woolford, J.L., Jr. and Gao, N. (2017) Structural snapshot of cytoplasmic pre-60S ribosomal particles bound by Nmd3, Lsg1, Tif6 and Reh1. *Nat. Struct. Mol. Biol.*, **24**, 214-220.
18. Klinge, S. and Woolford, J.L., Jr. (2019) Ribosome assembly coming into focus. *Nat. Rev. Mol. Cell. Biol.*, **20**, 116-131.

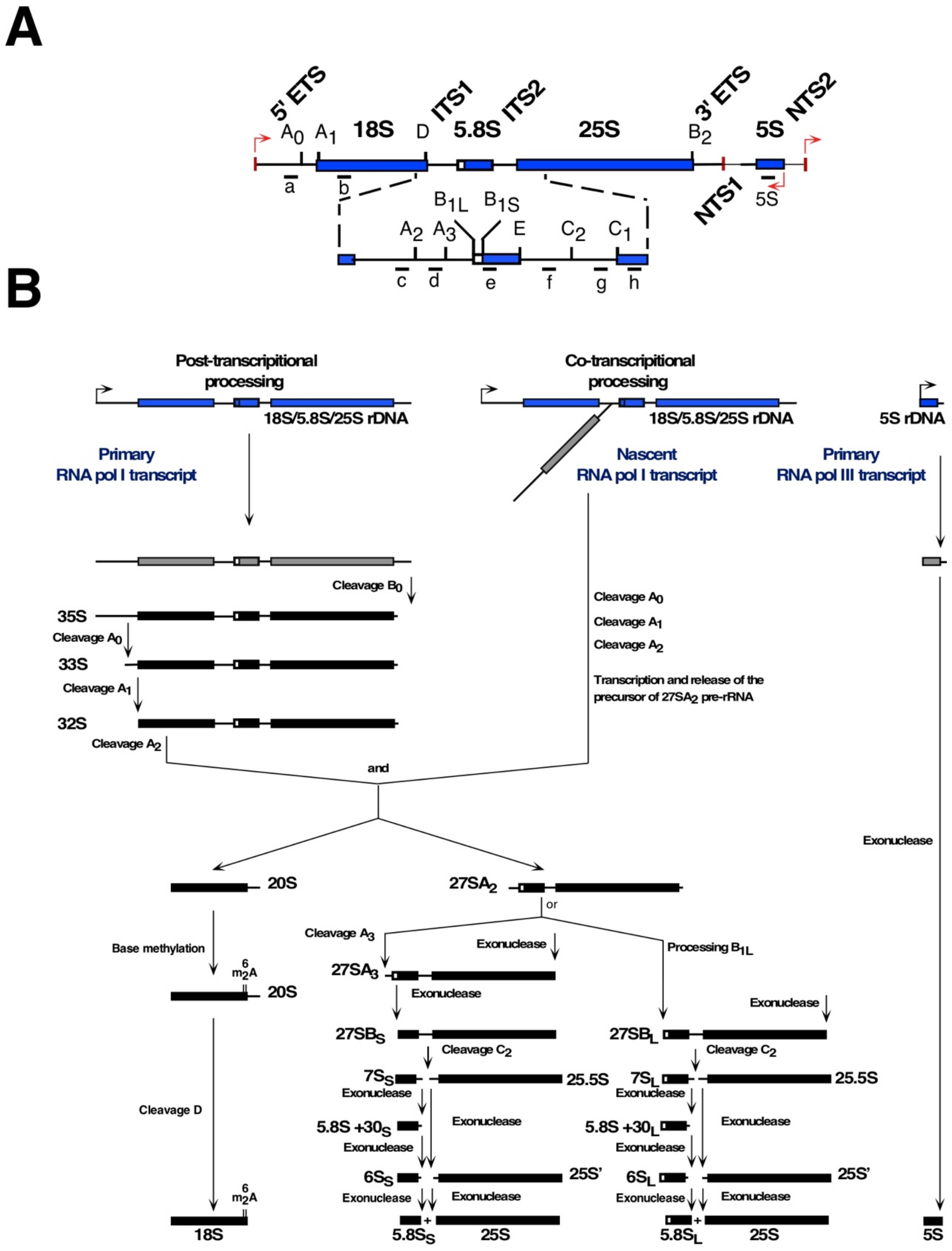
A
5' ETS
ITS1
ITS2
3' ETS
NTS2
A0
A1
18S
D
5.8S
25S
B2
5S
a
b
NTS1
5S
B1L
B1S
A2
A3
E
C2
C1
c
d
e
f
g
h
B
Post-transcripitional processing
Co-transcripitional processing
18S/5.8S/25S rDNA
18S/5.8S/25S rDNA
5S rDNA
Primary RNA pol I transcript
Nascent RNA pol I transcript
Primary RNA pol III transcript
Cleavage B0
35S
Cleavage A0
33S
Cleavage A1
32S
Cleavage A2
Cleavage A0
Cleavage A1
Cleavage A2
Transcription and release of the precursor of 27SA2 pre-rRNA
and
Exonuclease
20S
27SA2
or
Base methylation
Cleavage A3
Exonuclease
Processing B1L
m62A
20S
27SA3
Exonuclease
Exonuclease
27SBS
27SBL
Cleavage C2
Cleavage C2
Cleavage D
7SS
25.5S
7SL
25.5S
Exonuclease
Exonuclease
Exonuclease
Exonuclease
5.8S +30S
5.8S +30L
Exonuclease
Exonuclease
6SS
25S'
6SL
25S'
Exonuclease
Exonuclease
Exonuclease
Exonuclease
m62A
18S
5.8SS
25S
5.8SL
25S
5S

Figure S1

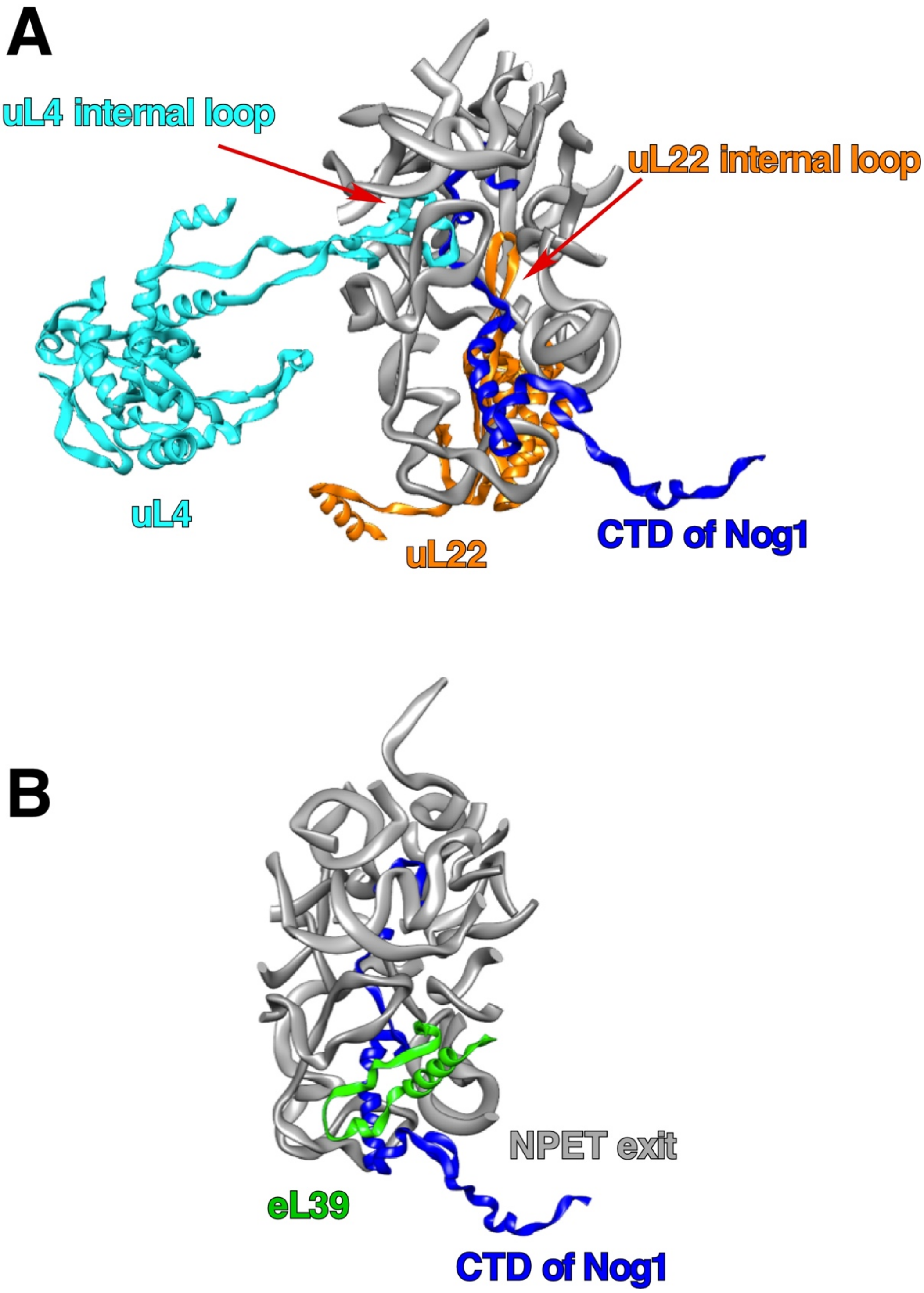
A
uL4 internal loop
uL22 internal loop
uL4
uL22
CTD of Nog1
B
NPET exit
eL39
CTD of Nog1

Figure S2

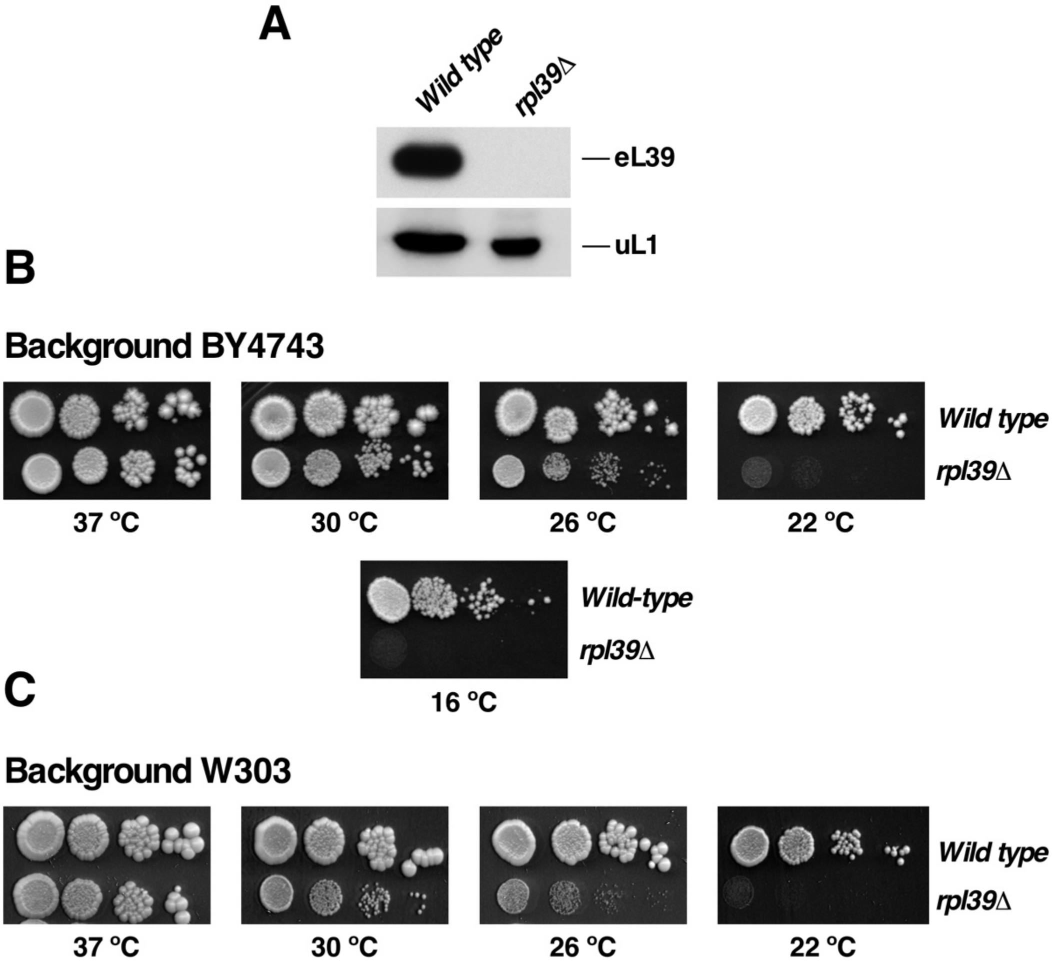
A
Wild type
rpl39Δ
eL39
uL1
B
Background BY4743
Wild type
rpl39Δ
37 °C
30 °C
26 °C
22 °C
Wild-type
rpl39Δ
16 °C
C
Background W303
Wild type
rpl39Δ
37 °C
30 °C
26 °C
22 °C

Figure S3

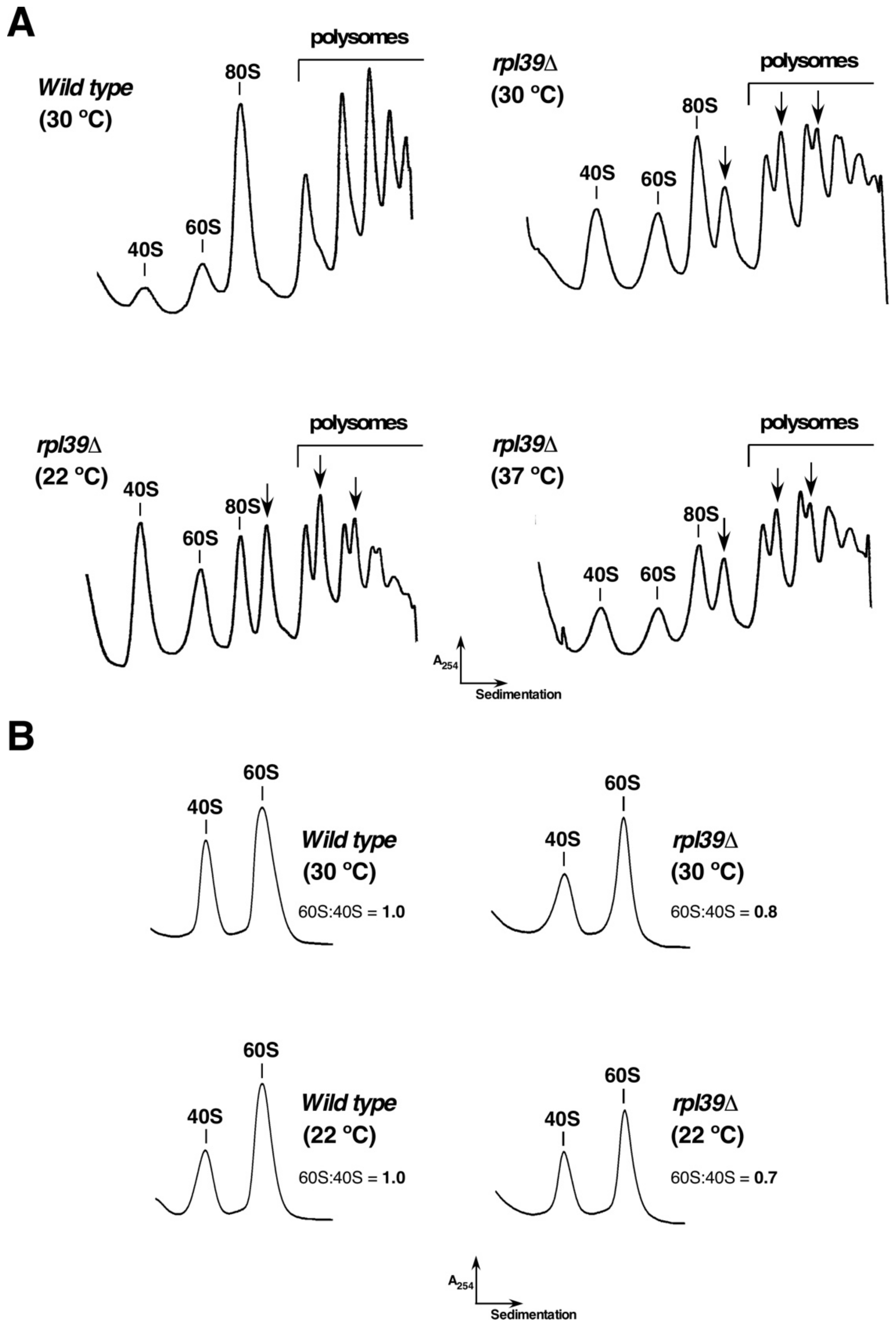
A
Wild type
(30 °C)
40S
60S
80S
polysomes
rpl39Δ
(30 °C)
40S
60S
80S
polysomes
rpl39Δ
(22 °C)
40S
60S
80S
polysomes
rpl39Δ
(37 °C)
40S
60S
80S
polysomes
A254
Sedimentation
B
40S
60S
Wild type
(30 °C)
60S:40S = 1.0
40S
60S
rpl39Δ
(30 °C)
60S:40S = 0.8
40S
60S
Wild type
(22 °C)
60S:40S = 1.0
40S
60S
rpl39Δ
(22 °C)
60S:40S = 0.7
A254
Sedimentation

Figure S4

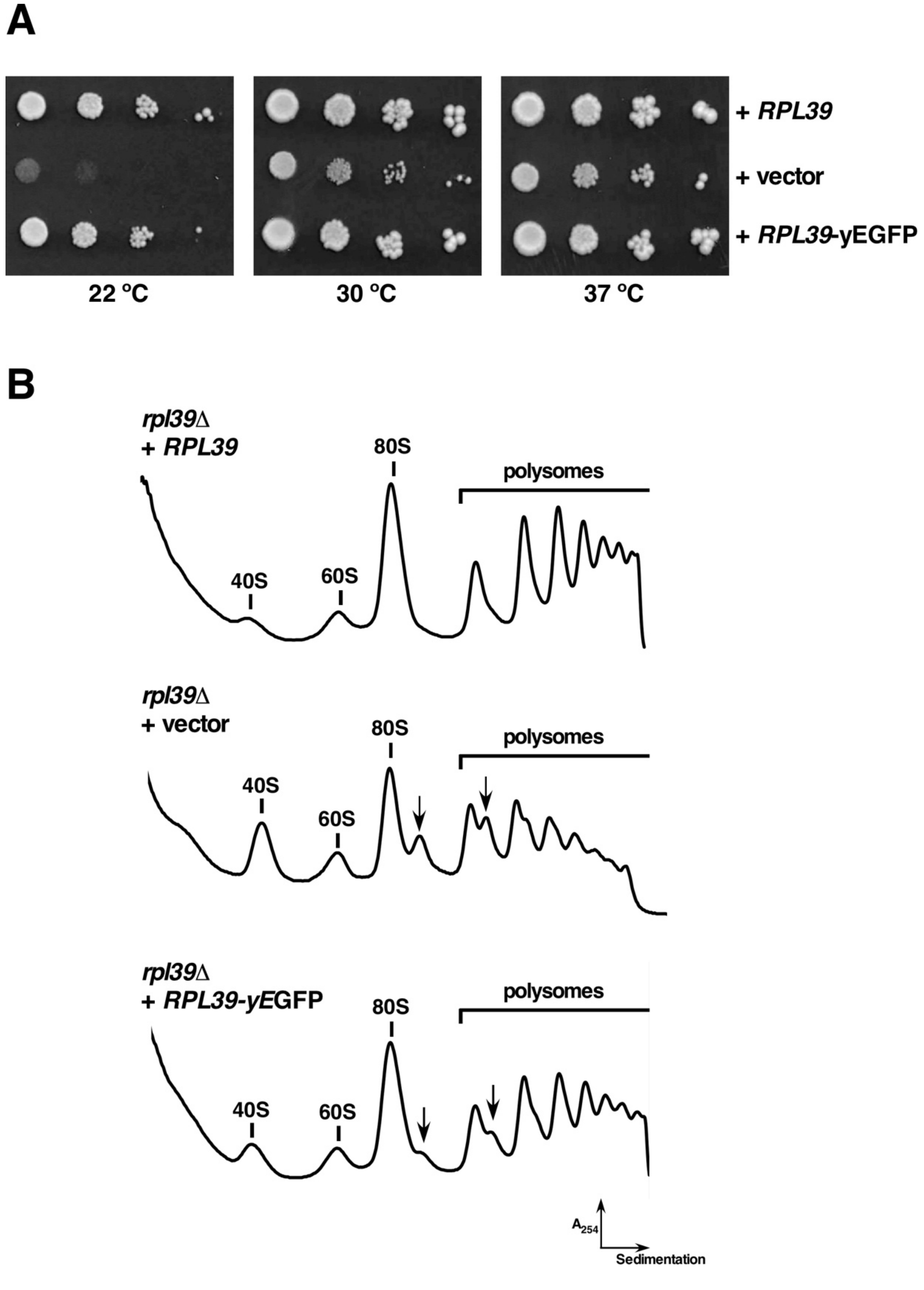
A
+ RPL39
+ vector
+ RPL39-yEGFP
22 °C
30 °C
37 °C
B
rpl39Δ
+ RPL39
80S
polysomes
40S
60S
rpl39Δ
+ vector
80S
polysomes
40S
60S
rpl39Δ
+ RPL39-yEGFP
80S
polysomes
40S
60S
A254
Sedimentation

Figure S5

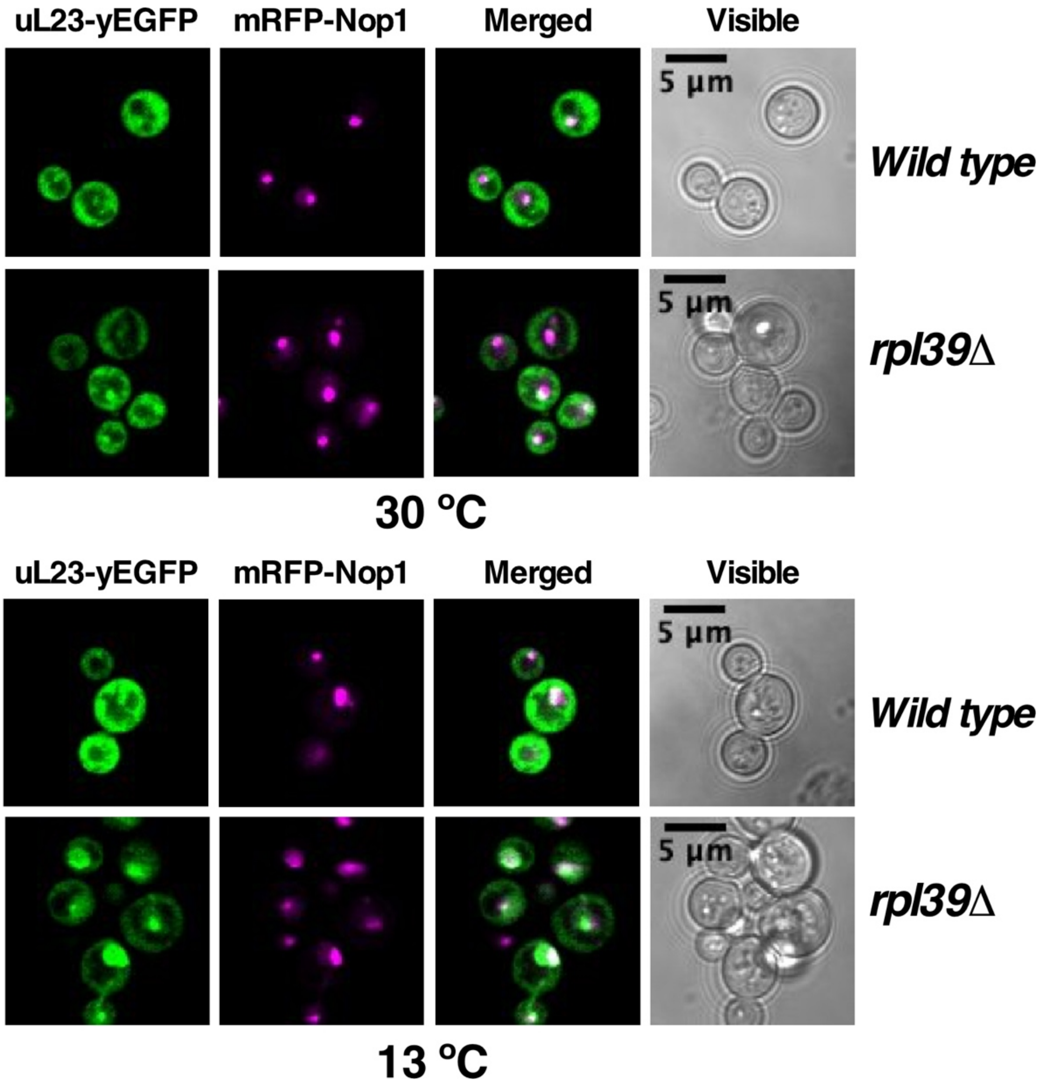
uL23-yEGFP
mRFP-Nop1
Merged
Visible
5 µm
Wild type
rpl39Δ
30 °C
13 °C

Figure S6

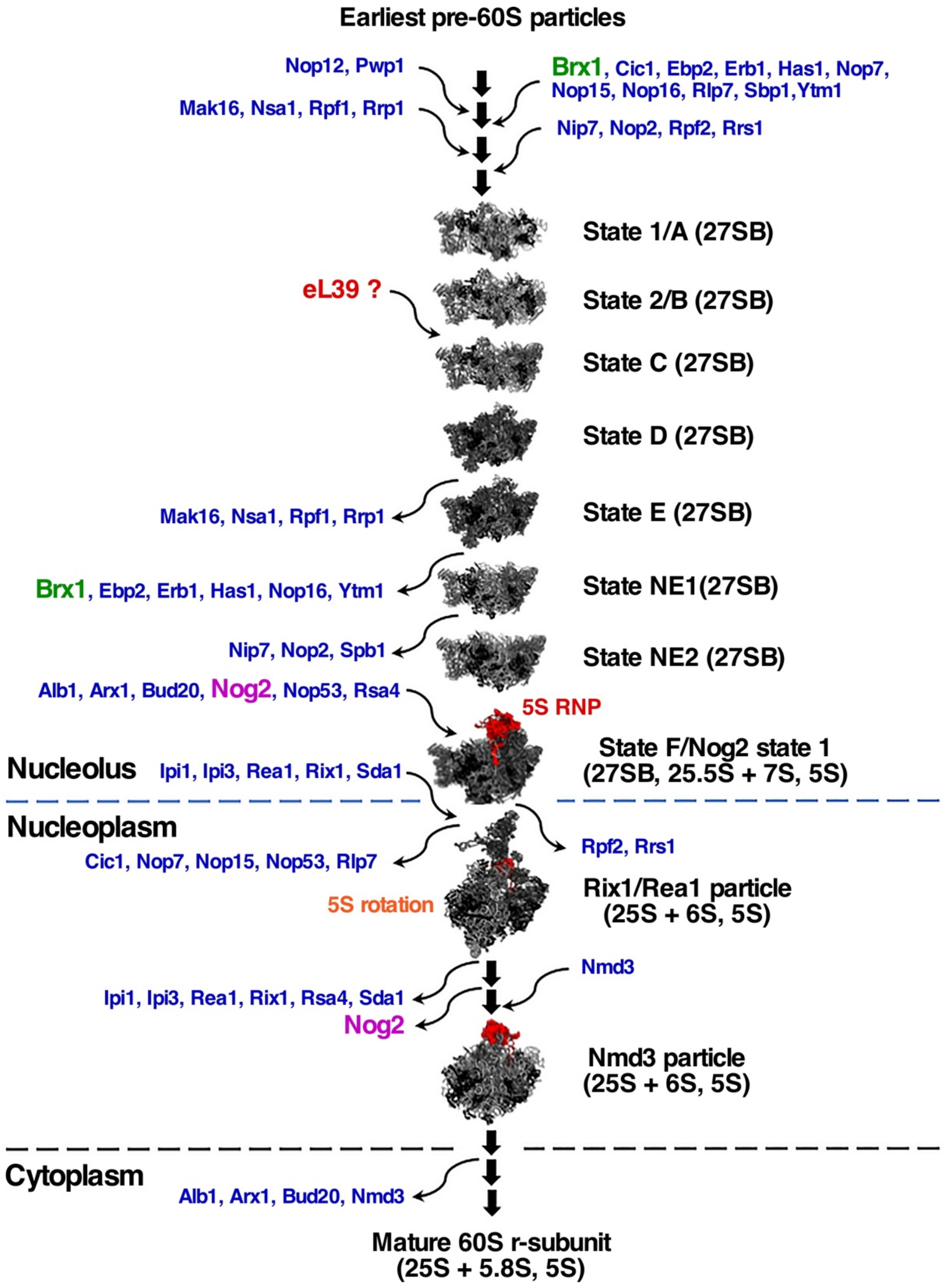
Earliest pre-60S particles
Nop12, Pwp1
Mak16, Nsa1, Rpf1, Rrp1
Brx1, Cic1, Ebp2, Erb1, Has1, Nop7,
Nop15, Nop16, Rlp7, Sbp1,Ytm1
Nip7, Nop2, Rpf2, Rrs1
State 1/A (27SB)
eL39 ?
State 2/B (27SB)
State C (27SB)
State D (27SB)
Mak16, Nsa1, Rpf1, Rrp1
State E (27SB)
Brx1, Ebp2, Erb1, Has1, Nop16, Ytm1
State NE1(27SB)
Nip7, Nop2, Spb1
State NE2 (27SB)
Alb1, Arx1, Bud20, Nog2, Nop53, Rsa4
5S RNP
State F/Nog2 state 1
(27SB, 25.5S + 7S, 5S)
Nucleolus
Ipi1, Ipi3, Rea1, Rix1, Sda1
Nucleoplasm
Cic1, Nop7, Nop15, Nop53, Rlp7
Rpf2, Rrs1
5S rotation
Rix1/Rea1 particle
(25S + 6S, 5S)
Ipi1, Ipi3, Rea1, Rix1, Rsa4, Sda1
Nog2
Nmd3
Nmd3 particle
(25S + 6S, 5S)
Cytoplasm
Alb1, Arx1, Bud20, Nmd3
Mature 60S r-subunit
(25S + 5.8S, 5S)

Figure S7

Densities for eL39 in state E particles

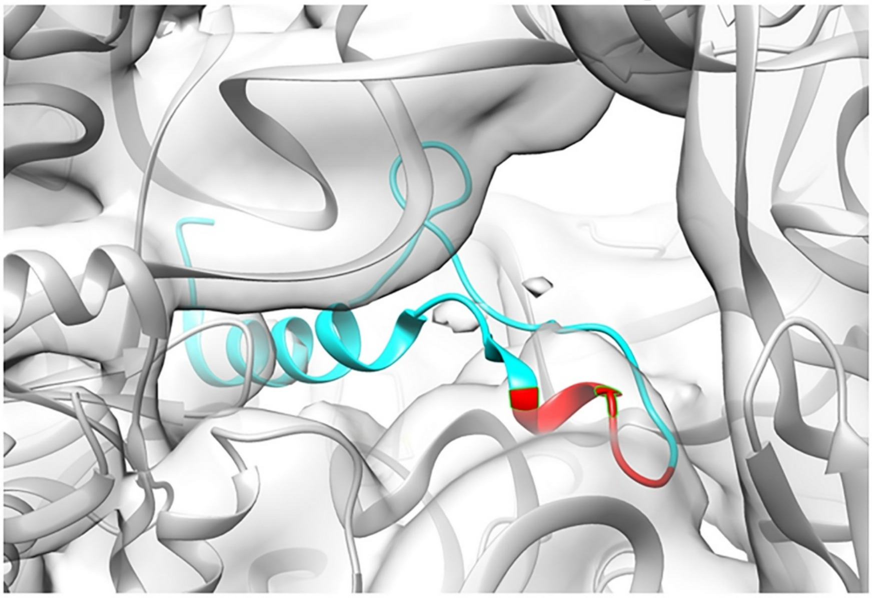

Densities for eL39 in Nog2 state 1 particles

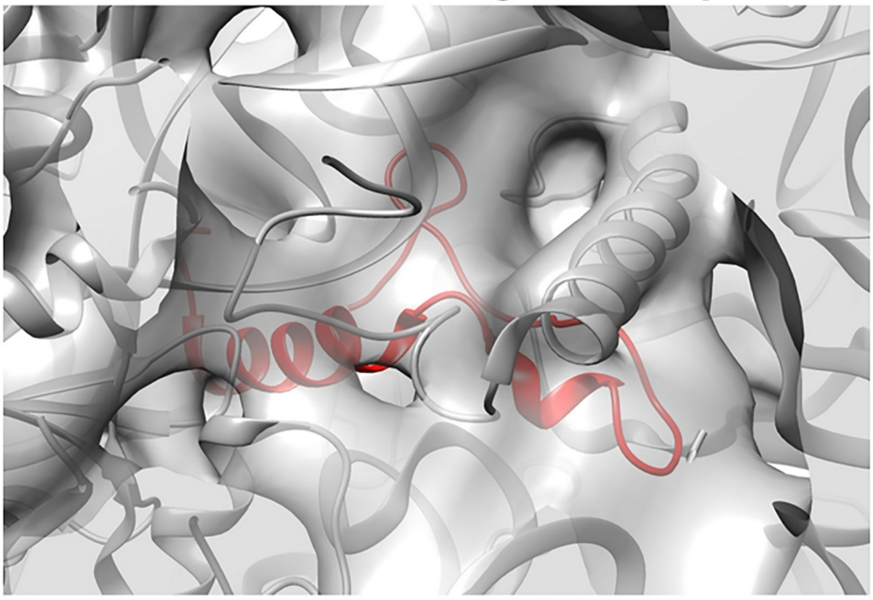

Figure S8

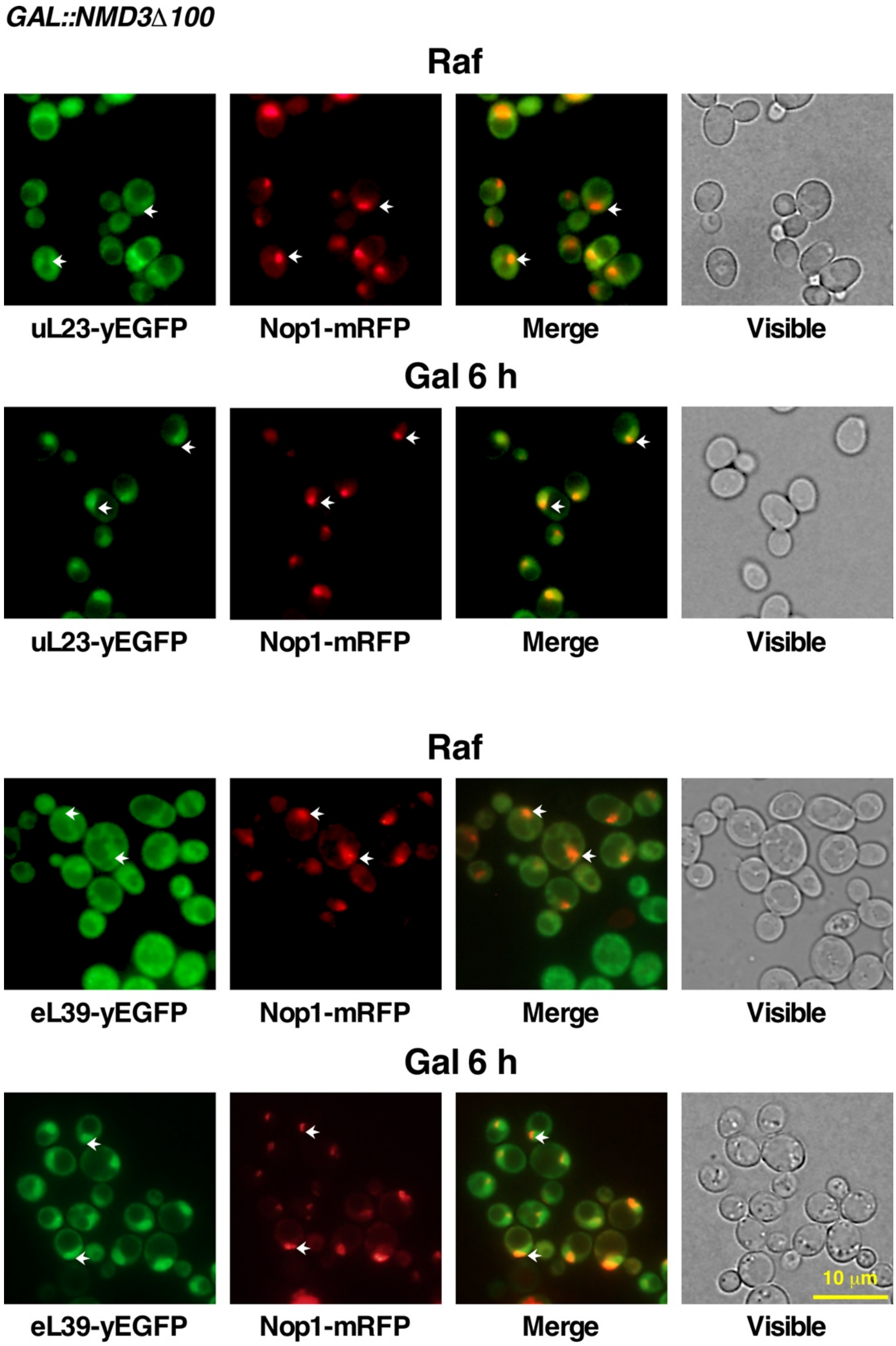
GAL::NMD3Δ100
Raf
uL23-yEGFP
Nop1-mRFP
Merge
Visible
Gal 6 h
uL23-yEGFP
Nop1-mRFP
Merge
Visible
Raf
eL39-yEGFP
Nop1-mRFP
Merge
Visible
Gal 6 h
eL39-yEGFP
Nop1-mRFP
Merge
Visible
10 μm

Figure S9

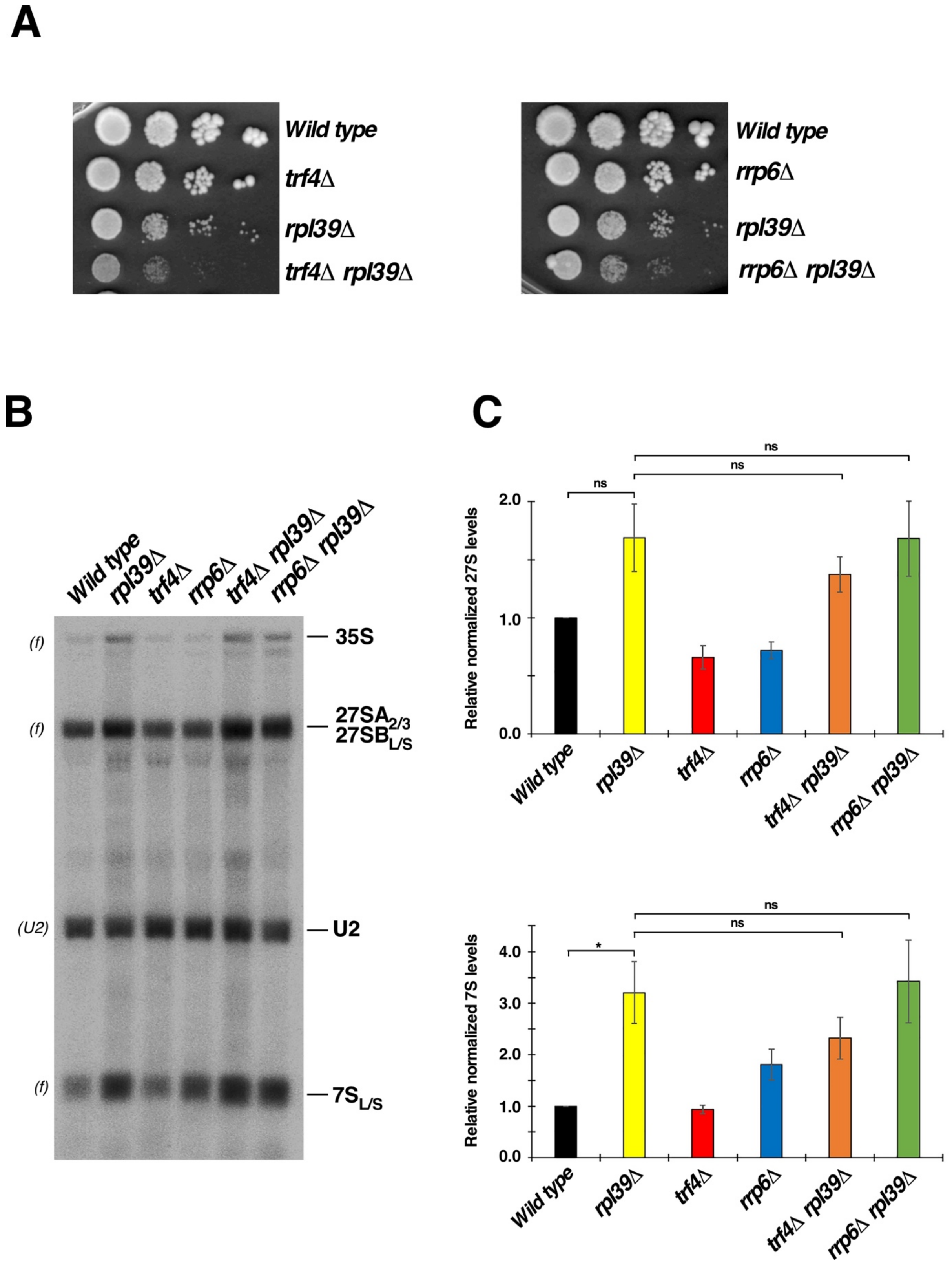
A
Wild type
trf4Δ
rpl39Δ
trf4Δ rpl39Δ
Wild type
rrp6Δ
rpl39Δ
rrp6Δ rpl39Δ
B
Wild type
rpl39Δ
trf4Δ
rrp6Δ
trf4Δ rpl39Δ
rrp6Δ rpl39Δ
(f)
35S
(f)
27SA2/3
27SBL/S
(U2)
U2
(f)
7SL/S
C
Relative normalized 27S levels
2.0
1.0
0.0
ns
ns
ns
Wild type
rpl39Δ
trf4Δ
rrp6Δ
trf4Δ rpl39Δ
rrp6Δ rpl39Δ
Relative normalized 7S levels
4.0
3.0
2.0
1.0
0.0
*
ns
ns
Wild type
rpl39Δ
trf4Δ
rrp6Δ
trf4Δ rpl39Δ
rrp6Δ rpl39Δ

Figure S10

rpf2Δ255-344 : wild type (NOG2-TAP)
Assembly factors

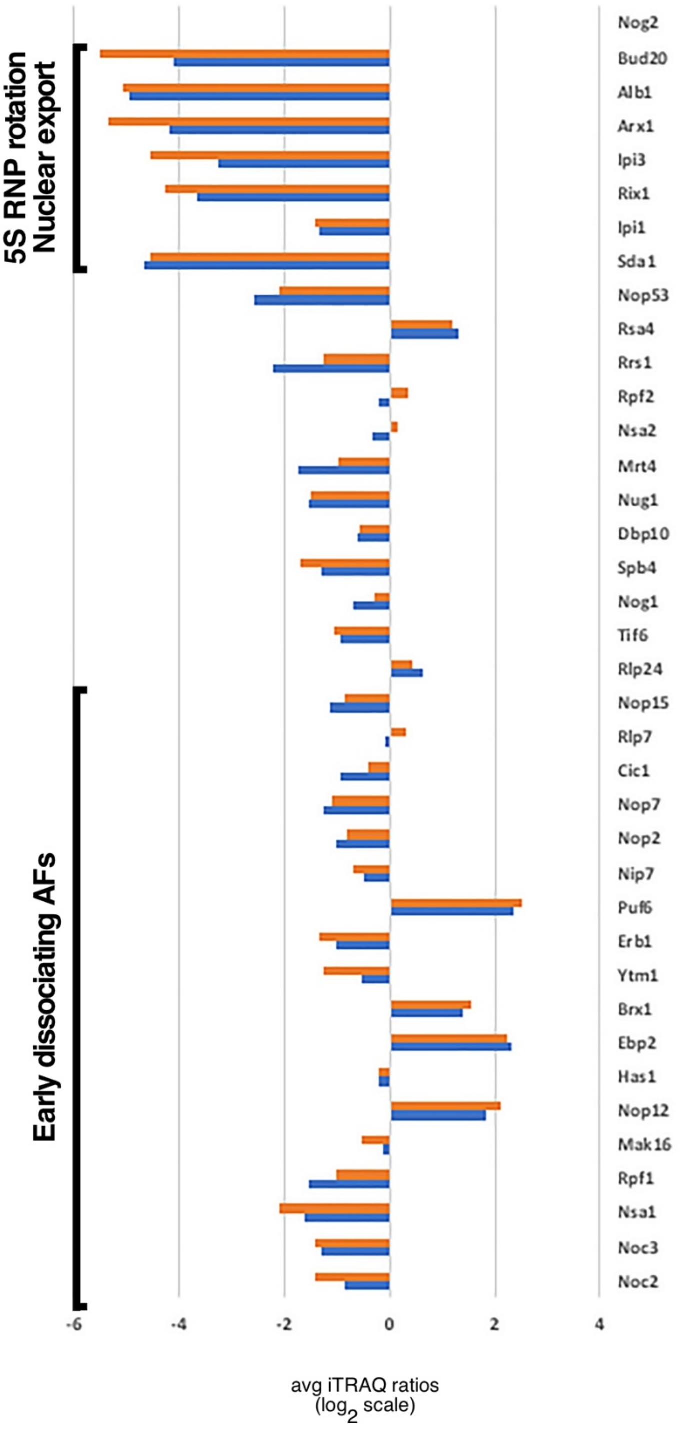

Figure S11
